# ASb_3_Mn_9_O_19_ (A = K or Rb): New Mn‐Based 2D Magnetoplumbites with Geometric and Magnetic Frustration

**DOI:** 10.1002/adma.202417906

**Published:** 2024-12-31

**Authors:** Jianyi Chen, Stuart Calder, Joseph A. M. Paddison, Gina Angelo, Liana Klivansky, Jian Zhang, Huibo Cao, Xin Gui

**Affiliations:** ^1^ Department of Chemistry University of Pittsburgh Pittsburgh PA 15260 USA; ^2^ Neutron Scattering Division Oak Ridge National Laboratory Oak Ridge TN 37831 USA; ^3^ The Molecular Foundry Lawrence Berkeley National Laboratory Berkeley CA 94720 USA

**Keywords:** 2‐D magnetic correlation, Kagome lattice, magnetic frustration, magnetoplumbites

## Abstract

Magnetoplumbites are one of the most broadly studied families of hexagonal ferrites, typically with high magnetic ordering temperatures, making them excellent candidates for permanent magnets. However, magnetic frustration is rarely observed in magnetoplumbites. Herein, the discovery, synthesis, and characterization of the first Mn‐based magnetoplumbite, as well as the first magnetoplumbite involving pnictogens (Sb), ASb_3_Mn_9_O_19_ (A = K or Rb) are reported. The Mn^3+^ (*S* = 2) cations, further confirmed by DC magnetic susceptibility and X‐ray photoelectron spectroscopy, construct three geometrically frustrated sublattices, including Kagome, triangular, and puckered honeycomb lattices. Magnetic properties measurements revealed strong antiferromagnetic spin–spin coupling as well as multiple low‐temperature magnetic features. Heat capacity data does not show any prominent λ‐anomaly, suggesting minimal associated magnetic entropy. Moreover, neutron powder diffraction (NPD) implied the absence of long‐range magnetic ordering in KSb_3_Mn_9_O_19_ down to 3 K. However, several magnetic peaks are observed in RbSb_3_Mn_9_O_19_ at 3 K, corresponding to an incommensurate magnetic structure. Interestingly, strong diffuse scattering is seen in the NPD patterns of both compounds at low angles and is analyzed by reverse Monte Carlo refinements, indicating short‐range spin ordering related to frustrated magnetism as well as 2D magnetic correlations in ASb_3_Mn_9_O_19_ (A = K or Rb).

## Graphical abstract

The first Mn‐based magnetoplumbites, ASb_3_Mn_9_O_19_ (A = K or Rb), are discovered, synthesized, and characterized. According to magnetic properties measurements, neutron diffraction, and reverse Monte Carlo refinement, short‐range magnetic correlations are observed in the new materials. These observations reveal 2D magnetic correlations and magnetic frustration in ASb_3_Mn_9_O_19_, making it a promising platform for inducing exotic quantum states.

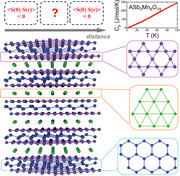

## 1 Introduction

Magnetic frustration originates from the competition between multiple magnetic exchange interactions, normally due to geometrically frustrated crystal lattices or chemical disorders.^[^ ^1^, ^2^, ^3^, ^4^, ^5^, ^6^, ^7^ ^]^ Geometrical magnetic frustration in quantum materials has drawn tremendous attention and is of great importance in the material chemistry/physics community. Many intriguing quantum states have been proposed/observed in a variety of material systems due to geometric frustration, e.g., spin liquid/quantum spin liquid^[^ ^8^, ^9^, ^10^, ^11^, ^12^, ^13^, ^14^ ^]^ and quantum spin ice.^[^ ^15^, ^16^, ^17^, ^18^ ^]^ Despite being investigated for decades, there still exists a long‐standing need for the discovery of new magnetically frustrated materials due to limitations in existing systems, e.g., chemical disorder that can lead to ambiguity in observing quantum spin liquid states.^[^ ^1^, ^2^, ^10^ ^]^ Chemical design plays a crucial role in expanding the pool of frustrated magnets while starting from specific magnetic crystal lattices is proven to be one of the most effective means to achieve such a goal. For instance, a variety of frustrated magnets with magnetic triangular,^[^ ^9^, ^12^, ^13^, ^19^, ^20^ ^]^ Kagome,^[^ ^21^, ^22^, ^23^ ^]^ honeycomb,^[^ ^24^, ^25^, ^26^, ^27^ ^]^ pyrochlore^[^ ^11^, ^16^, ^17^, ^18^ ^]^ and square net^[^ ^28^, ^29^ ^]^ lattices have been discovered and investigated. Ferrites with a spinel formula of AB_2_O_4_ and cubic symmetry, as one of the most well‐known and heavily studied families of magnetic materials, were explored for their high magnetic ordering temperatures and developed to serve as permanent magnets in many applications.^[^ ^30^, ^31^, ^32^ ^]^ Moreover, magnetic frustration has also been commonly observed in AB_2_O_4_ ferrites due to the existence of a pyrochlore lattice of B‐site ions, for instance, MCr_2_O_4_^[^ ^33^, ^34^ ^]^ and LiV_2_O_4_.^[^ ^35^, ^36^, ^37^ ^]^ Interestingly, when more chemical complexities are involved in AB_2_O_4_ ferrites, a higher structural/compositional tunability is induced, leaving numerous possibilities open to invent more magnetically frustrated quantum materials.

*M*‐type hexaferrites, also known as magnetoplumbites, are one of the most widely studied subgroups of AB_2_O_4_ ferrites, adopting a general formula of AB_12_O_19_.^[^ ^38^, ^39^ ^]^ Here, A is mainly alkali,^[^ ^40^, ^41^ ^]^ alkaline‐earth elements,^[^ ^42^, ^43^ ^]^ lanthanides,^[^ ^44^ ^]^ Pb^[^ ^45^ ^]^ or a mixture of them,^[^ ^46^, ^47^ ^]^ while B can be group 13 elements,^[^ ^48^, ^49^, ^50^ ^]^ transition metal elements including Ti, V, Cr, Fe, Co and Ni,^[^ ^45^, ^47^, ^51^, ^52^ ^]^ or a mixture.^[^ ^53^, ^54^, ^55^, ^56^ ^]^ They typically crystallize in a hexagonal unit cell with a space group of *P*6_3_/*mmc*, where A‐site ions are well separated by polyhedra formed by B and O and the cation B occupies various atomic sites. In terms of magnetic properties, magnetoplumbites with magnetic B cations are usually considered as great candidates for permanent magnets due to their high Curie temperatures.^[^ ^39^ ^]^ Interestingly, several sublattices of B can be found in AB_12_O_19_, e.g., triangular, Kagome and puckered honeycomb sublattices, which makes magnetoplumbites a promising material platform for inducing frustrated magnetism. However, only very limited examples of magnetoplumbites have been reported to show magnetic frustration, including spin glass in MCr_9p_Ga_12‐9p_O_19_ (M = Sr, Ba),^[^ ^57^, ^58^, ^59^, ^60^, ^61^, ^62^, ^63^, ^64^ ^]^ 2D magnetic frustration in LnMgAl_11_O_19_ (Ln = Pr, Nd) and LnZnAl_11_O_19_ (Ln = Pr, Nd, Sm, Eu, Gd, Tb),^[^ ^65^, ^66^ ^]^ spin‐glass state in SrCo_6_Ti_6_O_19_^[^ ^67^, ^68^ ^]^ and BaFe_12_O_19_,^[^ ^69^ ^]^ as well as a large frustration factor of ≈26 observed in BaSn_6_Co_6_O_19_.^[^ ^53^ ^]^

Here, we present the discovery and characterization of a novel type of magnetoplumbite, ASb_3_Mn_9_O_19_ (A = K or Rb). To the best of our knowledge, they are the first Mn‐based magnetoplumbites, as well as the first magnetoplumbites involving pnictogens (Sb). Polycrystalline samples were synthesized and characterized, and they both adopt a magnetoplumbite structure. According to the single crystal X‐ray diffraction (XRD), we determined that there are three distinct Mn sites in ASb_3_Mn_9_O_19_, forming a Kagome, a puckered honeycomb, and a triangular sublattice, respectively. The magnetic properties and heat capacity measurements reveal several low‐temperature magnetic features down to 1.8 K. The Curie–Weiss (CW) fitting on the DC magnetic susceptibility shows strong antiferromagnetic coupling between Mn^3+^ (*S* = 2), while the single valency and trivalent nature of Mn are consistent with the X‐ray photoelectron spectroscopy (XPS) results. Neutron powder diffraction (NPD) further confirms the absence of long‐range ordering in KSb_3_Mn_9_O_19_ but indicates the possible incommensurate magnetic ordering of RbSb_3_Mn_9_O_19_. We also observed strong diffuse scattering in NPD patterns in both KSb_3_Mn_9_O_19_ and RbSb_3_Mn_9_O_19_, which likely originates from frustrated magnetism as well as 2D magnetic correlations. The discovery of the new insulating ASb_3_Mn_9_O_19_, as the first Mn‐based magnetoplumbite, provides a great platform for investigating frustrated magnetism in the puckered honeycomb, Kagome, and triangular sublattices, as well as the intertwining properties among them. Additionally, it allows further modification of the magnetic sites, suggesting the potential for discovering more exotic quantum states, such as new integer‐spin‐frustrated magnets.^[^ ^70^, ^71^, ^72^, ^73^ ^]^ New quantum spin liquids may also be realized in this system if a *S* = ½ spin state can be achieved.

## 2 Results and Discussion

### 2.1 Crystal Structure of ASb_3_Mn_9_O_19_

**Figure 1:**
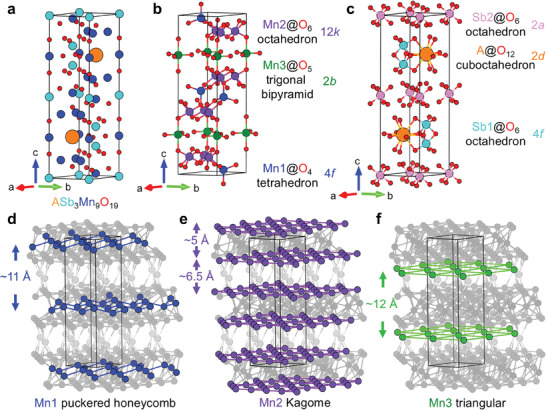
a) Crystal structure of ASb_3_Mn_9_O_19_ (A = K or Rb) where orange, cyan, blue, and red spheres represent A, Sb, Mn, and O atoms, respectively. b) Coordination types of Mn1, Mn2, and Mn3 sites where blue, purple, and green spheres stand for Mn1, Mn2, and Mn3 sites, respectively. c) Coordination types of A, Sb1, and Sb2 sites, are represented by orange, cyan, and pink spheres. d–f) Sublattices of Mn1, Mn2, and Mn3 sites with each site are highlighted in the crystal structure as well as the separation of neighboring planes of each site.

Crystal structures of ASb_3_Mn_9_O_19_ (A = K and Rb) are found to be similar, both of which crystallize in a hexagonal space group *P*6_3_/*mmc* (No. 194). The crystallographic data including refined anisotropic displacement parameters and equivalent isotropic thermal displacement parameters of both compounds, are summarized in **Table** **1** and Tables S1 and S2 (Supporting Information). The crystal structure of ASb_3_Mn_9_O_19_ is similar to the well‐known structural family, *M*‐type hexaferrite, or magnetoplumbite. Therefore, ASb_3_Mn_9_O_19_ becomes the first example of a Mn‐based magnetoplumbite as well as the first magnetoplumbite involving pnictogens (Sb). The crystal structure of ASb_3_Mn_9_O_19_ is shown in **Figure** **1**a while the coordination of metal cations is illustrated in Figure 1b,c. As seen in Figure 1b, three crystallographically distinct Mn sites can be found in ASb_3_Mn_9_O_19_, namely Mn1 (Wyck. 4*f*), Mn2 (Wyck. 12*k*), and Mn3 (Wyck. 2*b*) sites. Each Mn site coordinates differently with oxygen atoms, i.e., Mn1, Mn2, and Mn3 occupy the center of Mn1@O_4_ tetrahedron, Mn2@O_6_ octahedron, and Mn3@O_5_ trigonal bipyramid, respectively. Meanwhile, Sb atoms occupy two crystallographic sites, Sb1 (Wyck. 4*f*) and Sb2 (Wyck. 2*a*) sites, and A possesses only one atomic site (Wyck. 2*d*). Both Sb sites are 6‐coordinated with oxygen, building Sb@O_6_ octahedra, while A coordinates with 12 oxygen atoms, constructing A@O_12_ cuboctahedra, as shown in Figure 1c. Moreover, Mn atoms show an interesting framework, as can be seen in Figure 1d–f. Each crystallographically different Mn site forms a distinct Mn sublattice, Mn1 puckered honeycomb, Mn2 Kagome and Mn3 triangular sublattices. The separation between each Mn sheet is also shown in Figure 1–f. A well‐separated puckered honeycomb sublattice of Mn1 and a well‐separated triangular Mn3 sublattice can be found. The Kagome lattice of Mn2 can be treated as a “quasi‐bilayer Kagome” with a separation of ≈5 Å while each “bilayer” is moderately separated by ≈6.5 Å for both materials. Additionally, it is noteworthy that the Mn2–Mn2 bond length within the Kagome sublattice is the shortest Mn–Mn distance (3.030 (1) Å for K and 3.041 (1) Å for/ Rb) in both compounds. Therefore, one can expect that the primary magnetic correlations will take place within the Kagome layers, as further detailed later in the DC magnetic and NPD measurements.

**Table 1:** Single crystal structure refinement for ASb_3_Mn_9_O_19_ (A = K or Rb).

Refined formula	KSb_2.82(1)_Mn_9.18(1)_O_19_	RbSb_2.93(1)_Mn_9_O_19_
Temperature (K)	293 (2)	293 (2)
F.W. (g/mol)	1190.45	1240.66
Space group; Z	*P*6_3_/mmc; 2	*P*6_3_/mmc; 2
*a*(Å)	6.0606 (1)	6.0818 (7)
*c*(Å)	23.853 (1)	23.905 (4)
V (Å^3^)	758.76 (5)	765.8 (2)
θ range (°)	3.416–33.191	3.409–34.343
No. reflections; *R_int_* No. independent reflections No. parameters	21526; 0.0631	21395; 0.0401
	620	681
	32	34
*R_1_: ωR_2_* (*I*>2δ(*I*))	0.0304; 0.0657	0.0291; 0.0594
Goodness of fit	1.199	1.387
Diffraction peak and hole (e^−^/ Å^3^)	1.308; −1.268	1.174; −0.892

The potential superexchange pathways are illustrated in Figure S1, (Supporting Information). As can be seen, for Mn1–Mn1 magnetic interaction, Mn1‐O‐Sb2‐O‐Mn1, and Mn1‐O‐Mn2‐O‐Mn1 are two possible pathways due to the corner‐sharing nature of Mn1@O_4_ tetrahedron and Sb2/Mn2@O_6_ octahedron. Because of the edge‐sharing feature of Mn2@O_6_ octahedra, the most plausible superexchange pathway is Mn2‐O‐Mn2. Furthermore, Mn3–Mn3 magnetic interactions are mediated by Mn3‐O‐Sb1‐O‐Mn3 pathway, due to the corner‐sharing Mn3@O_5_ trigonal bipyramid and Sb1@O_6_ octahedron. Due to the fact that Mn1@O_4_, Mn2@O_6,_ and Mn3@O_5_ polyhedra are mutually corner‐sharing, potential interlayer magnetic exchange interaction can also be proposed through a Mn1‐O‐Mn2‐O‐Mn3 superexchange pathway.

### 2.2 Determination of Chemical Composition and Chemical Disorder

Based on the single crystal XRD results, a chemical disorder or vacancy can be assigned to the Sb2 site in ASb_3_Mn_9_O_19_. If the Sb2 site was treated as a fully occupied ordered site, the crystallographic refinement results worsen and are not acceptable for both materials; see Tables S3,S4 (Supporting Information). When the Sb2 site occupancy is relaxed in refinement, 10 (1)% and 7 (1)% vacancies were found for KSb_3_Mn_9_O_19_ and RbSb_3_Mn_9_O_19_, respectively, therefore leading to formulas of KSb_2.90(1)_Mn_9_O_19_ and RbSb_2.93(1)_Mn_9_O_19_. Due to a smaller electron count of Mn than Sb, it is possible that partial Mn occupancy exists in the Sb2 site, resulting in formulas of KSb_2.82(1)_Mn_9.18(1)_O_19_ and RbSb_2.87(1)_Mn_9.13(1)_O_19_.

**Figure 2:**
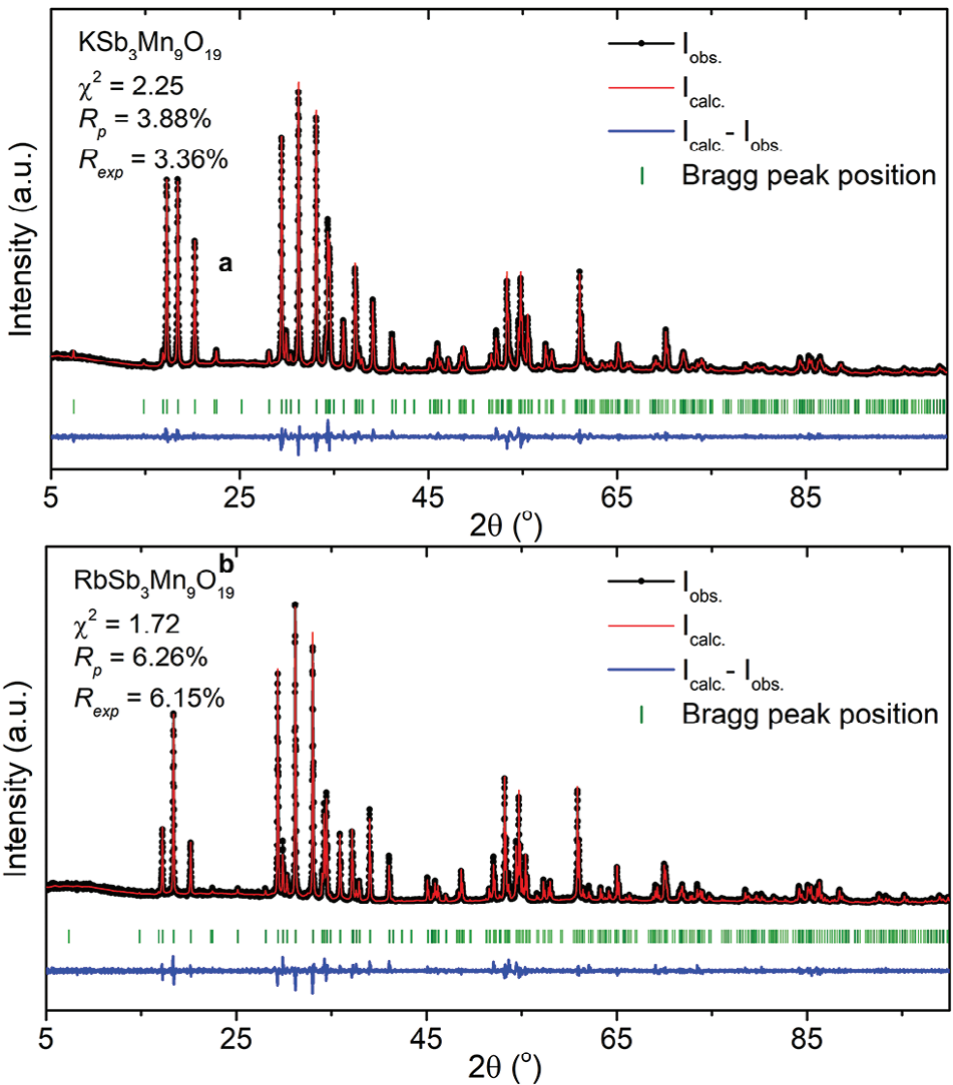
Powder XRD patterns with Rietveld refinement of a) KSb_3_Mn_9_O_19_ and b) RbSb_3_Mn_9_O_19_.RbSb_3_Mn_9_O_19_ in the rest of this study.

To determine accurate chemical compositions, SEM‐EDS measurements were performed on multiple pieces of each sample. The EDS results are summarized in Tables S5 and S6 (Supporting Information). For KSb_3_Mn_9_O_19_, the EDS determines the composition to be K_1.00(3)_Sb_2.96(6)_Mn_9.44(7)_O_19_, while for Rb compound it is Rb_1.0(1)_Sb_3.16(7)_Mn_9.49(6)_O_19_. The oxygen contents are not included due to its low electron count, and thus cannot be accurately determined by EDS. Considering that the sample is semiconducting, as described later in Section 2.9, electrons from the incident beam can be trapped on the sample surface, leading to a change in the effective energy of the primary electron beam. Therefore, the EDS compositions are within the error from the formulas determined by single crystal XRD. Moreover, the Mn/Sb ratio from EDS can help to confirm whether the Sb2 site is disordered with Mn or partially vacant. From Tables S5 and S6 (Supporting Information), Mn/Sb ratios were found to be 3.19 (9) in KSb_3_Mn_9_O_19_ and 3.00 (9) in RbSb_3_Mn_9_O_19_. By comparing them to the Mn/Sb ratios determined by single crystal XRD, as shown in Table S7 (Supporting Information), we conclude that the formulas should be KSb_2.82(1)_Mn_9.18(1)_O_19_ and RbSb_2.93(1)_Mn_9_O_19_, determined by single crystal XRD, which are utilized in all the analysis below. The reason for using single‐crystal‐XRD formulas is the electron trapping effect in EDS for semiconducting samples, as stated before, which can lead to inaccuracy of the absolute compositions. Thus, Mn is mixed with Sb on the Sb2 site when A = K while Sb2 site is partially vacant in A = Rb in ASb_3_Mn_9_O_19_. For clarity, we will still denote the compounds as KSb_3_Mn_9_O_19_ and **Figure** **2**. Powder XRD patterns with Rietveld refinement of a. KSb3Mn9O19 and b. RbSb3Mn9O19.

Lastly, the charge is not balanced in ASb_3_Mn_9_O_19_ if the oxidation state of 3+ is assumed for both Sb, due to the starting material of Sb_2_O_3_, and Mn, evidenced by magnetic properties measurements and XPS results described later. There are two possible reasons for the unbalanced charge: Sb possesses two oxidation states, 3+ and 5+; there are oxygen vacancies in ASb_3_Mn_9_O_19_ if both Sb and Mn are trivalent.

### 2.3 Analysis of Phase Purity

The phase purity of polycrystalline ASb_3_Mn_9_O_19_ was determined by powder XRD via Rietveld refinement, as shown in Figure 2a,b. The χ^2^ was fit to be 2.25 and 1.72 while R_p_ and R_wp_ were found to be lower than 4% and 6.5% for KSb_3_Mn_9_O_19_ and RbSb_3_Mn_9_O_19_, respectively. The small difference between the observed and calculated patterns, together with the fitting parameters, imply that both materials are of high purity and that the crystal structure from single crystal XRD is consistent with powder XRD. Moreover, no prominent impurity peaks can be observed. The fitted lattice parameters are *a* = 6.06667 (1) Å and *c* = 23.89518 (9) Å for KSb_3_Mn_9_O_19_, and *a* = 6.08206 (1) Å and *c* = 23.90777 (9) Å for RbSb_3_Mn_9_O_19_. The slight expansion of unit cells observed in the powder XRD results, compared to those from single crystal XRD, is attributed to the elevated temperature in the sample chamber of the Bruker D2 PHASER we utilized (≈40 °C).

### 2.4 Magnetic Properties

**Figure 3:**
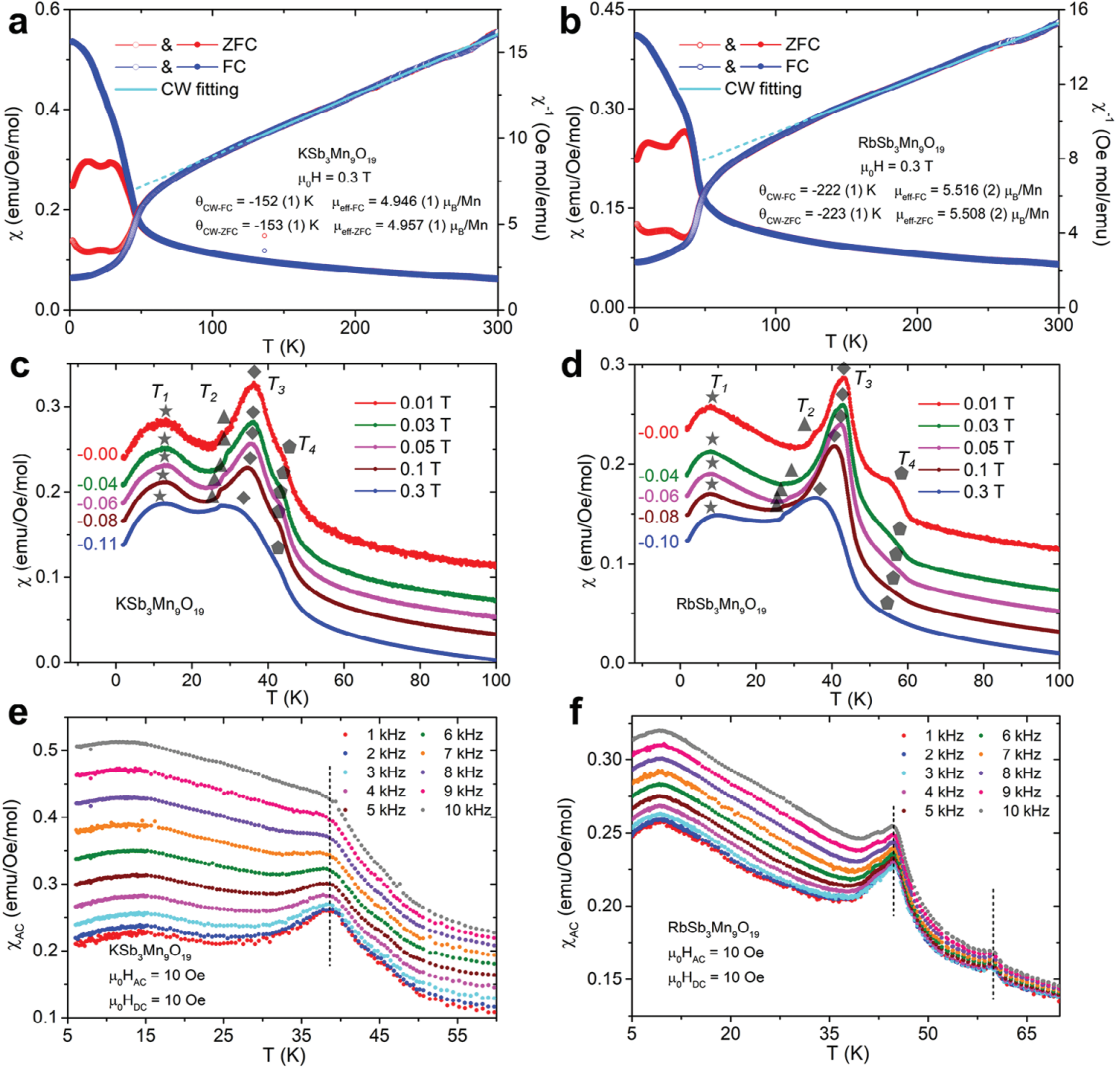
Temperature‐dependent magnetic susceptibility (χ) and its inverse (χ^−1^) measured under an external magnetic field of 0.3 T of a) KSb_3_Mn_9_O_19_ and b) RbSb_3_Mn_9_O_19_. Curie–Weiss fitting curves are shown in cyan. c,d) show the exploded view of χ under different magnetic fields for KSb_3_Mn_9_O_19_ and RbSb_3_Mn_9_O_19_. The curves were offset to ensure clarity. AC magnetic susceptibility (χ_AC_) is measured under a DC magnetic field of 10 Oe and an AC magnetic field of 10 Oe with a variety of frequencies for e) KSb_3_Mn_9_O_19_ and f) RbSb_3_Mn_9_O_19_. The black dashed lines are for eye guide only.

Polycrystalline samples directly from the reaction crucibles were used to measure the magnetic properties of both KSb_3_Mn_9_O_19_ and RbSb_3_Mn_9_O_19_. **Figure** **3**a,b exhibit the temperature‐dependent magnetic susceptibility (χ) from 2 to 300 K under an external magnetic field of 0.3 T using zero‐field‐cooling (ZFC) and field‐cooling (FC) protocols. For both materials, an increase at ≈50 K is seen in both ZFC and FC curves while the ZFC and FC curves overlap at higher temperatures. At low temperatures, the two curves deviate at ≈42 K where the FC curves keep increasing and do not reach a plateau for both samples. By plotting the temperature‐dependent inverse magnetic susceptibility (χ^−1^), CW fitting is applied to both materials under ZFC and FC protocols from 125 to 300 K by using the formula

(1) $$\chi =\frac{C}{T-\theta_{\mathrm{CW}}}\;$$

where C is a temperature‐independent constant and is related to the effective moment (*µ*_eff_) via $\mu_{eff}=\sqrt{8C}$, and θ_CW_ is the CW temperature. The fitted parameters are similar between ZFC and FC modes. For KSb_3_Mn_9_O_19_, θ_CW_ is fitted to be −152 (1) K (ZFC) and −153 (1) K (FC) while *µ*_eff_ is 4.946 (1) *µ*_B_/Mn (ZFC) and 4.957 (1) *µ*_B_/Mn (FC). For RbSb_3_Mn_9_O_19_, significantly larger θ_CW_ and *µ*_eff_ are obtained. The θ_CW_ is −222 (1) K (ZFC) and −223 (1) K (FC) while *µ*_eff_ is 5.516 (2) *µ*_B_/Mn (ZFC) and 5.508 (2) *µ*_B_/Mn (FC). According to the fitted θ_CW_, antiferromagnetic interactions can be expected within the fitted temperature range. The deviation between the CW fitting lines and the observed data can be seen at ≈100 K for KSb_3_Mn_9_O_19_ and at ≈130 K for RbSb_3_Mn_9_O_19_. Based on the fitted ɛ_eff_, the average oxidation state of Mn in both materials is close to Mn^3+^ (*S* = 2), for which the spin‐only moment is ≈4.90 _B_. The larger *µ*_eff_ for RbSb_3_Mn_9_O_19_ can result from unaccounted contributions from orbital angular momentum. The trivalent Mn in both compounds is also consistent with the XPS results, as detailed later in Section 2.5. Moreover, the large antiferromagnetic θ_CW_ and the absence of long‐range magnetic ordering above 50 K in both compounds imply the existence of magnetic frustration.

To better investigate the low‐temperature behaviors of ASb_3_Mn_9_O_19_, Figure 3c,d show the ZFC curves measured under different external magnetic fields. The curves are offset vertically for clarity. Four kinks/peaks below 60 K can be seen in both compounds, denoted T_1_, T_2_, T_3,_ and T_4_, and are marked using distinct shapes. The position of kinks/peaks are found to be decreasing with increasing magnetic field, consistent with antiferromagnetic interactions based on fitted θ_CW_. Considering the broad peaks corresponding to T_1_ and T_3_ and the deviation of ZFC and FC peaks shown in Figure 3a,b, the temperature‐dependent AC magnetic susceptibility (χ_AC_) was measured for ASb_3_Mn_9_O_19_. The measurements were performed under various AC frequencies ranging from 1 to 10 kHz with applied DC and AC magnetic fields, both of which are 10 Oe. As illustrated in Figure 3e, only two broad peaks can be observed in χ_AC_ for KSb_3_Mn_9_O_19_, including peaks at ≈14 and ≈38 K, corresponding to T_1_ and T_3_. However, for RbSb_3_Mn_9_O_19_, an extra peak corresponding to T_4_ can be found such that three groups of peaks are seen at ≈9, ≈45 and ≈60 K. None of the peaks observed in either material was found to shift toward higher temperatures with larger AC frequencies, implying that the low‐temperature kinks/peaks observed in ASb_3_Mn_9_O_19_ do not originate from conventional spin‐glass state. The fact that the FC curves in both compounds do not reach a plateau below the deviation point under any applied magnetic field below 0.3 T also implies the lack of the conventional spin‐glass behavior, as can be seen in Figure S2, (Supporting Information).

**Figure 4:**
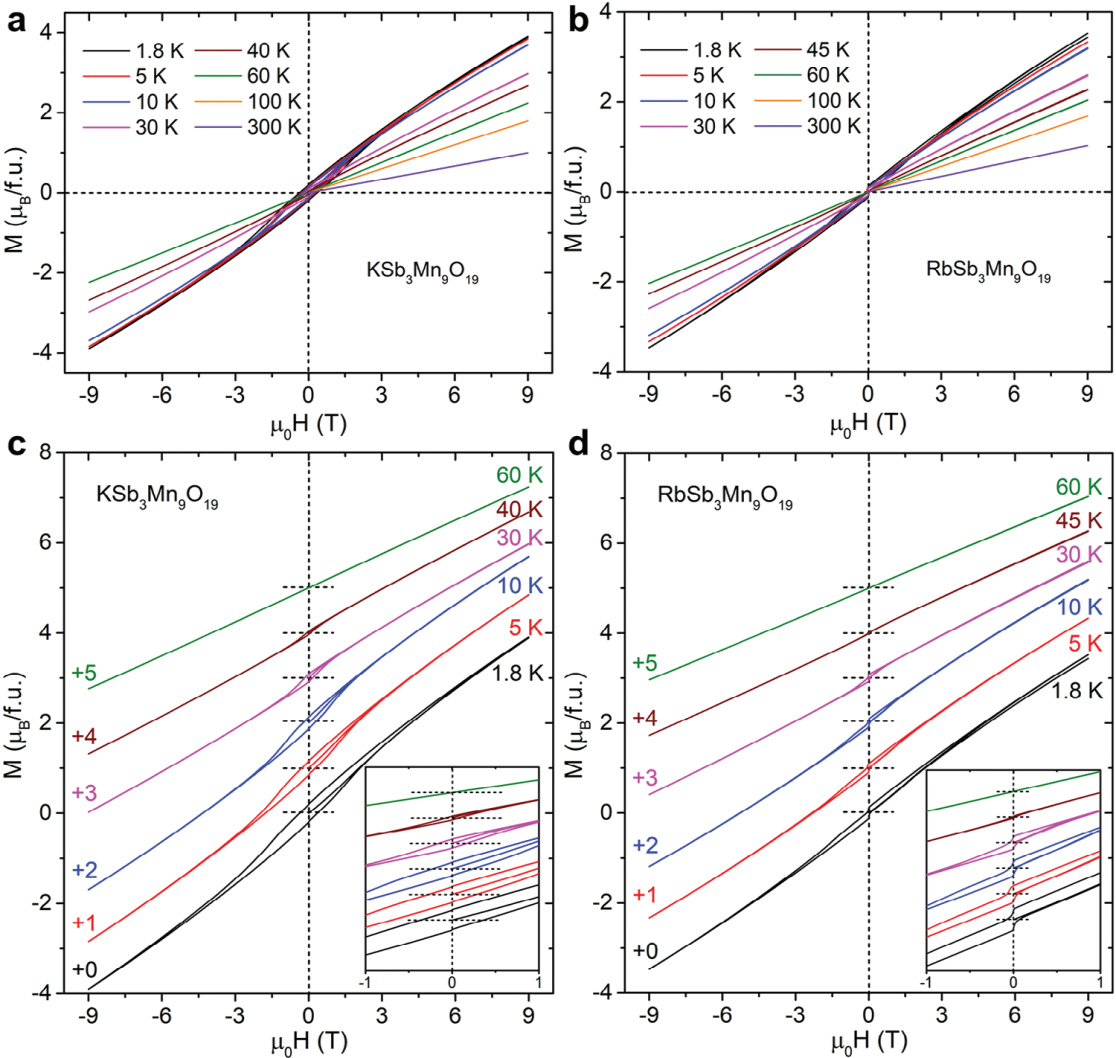
Magnetic hysteresis loops of a) KSb_3_Mn_9_O_19_ and b) RbSb_3_Mn_9_O_19_ was measured under different temperatures. Exploded view of hysteresis loops measured below 60 K between −9 and 9 T for c) KSb_3_Mn_9_O_19_ and d) RbSb_3_Mn_9_O_19_. The loops are offset for clarity. The insets of c,d. are the zoom‐ins of the main panel between −1 and 1 T.

The hysteresis loops of ASb_3_Mn_9_O_19_ are measured between −9 and 9 T under different temperatures and are plotted in **Figure** **4**a,b. The magnetization does not exhibit saturation at 9 T. The magnetic moments at 9 T and 1.8 K for KSb_3_Mn_9_O_19_ (µ_9*T*,1.8*K* − *K*_) are determined to be *µ*_9*T*,1.8*K* − *K*_ = 3.89 *µ*_B_/f.u., i.e., 0.42 *µ*_B_/Mn, which is only ≈ 9% of the fitted *µ*_eff_. Similar behavior can be seen in RbSb_3_Mn_9_O_19_ for which *µ*_9*T*,1.8*K* − *Rb*_ = 3.51 *µ*_B_/f.u., i.e., 0.39 *µ*_B_/Mn, which is ≈ 7% of the fitted *µ*_eff_. With increasing temperature, *µ*_9*T*,1.8*K*_ drops for both materials. Magnetic hysteresis is observed for both compounds under low temperatures, as shown in Figure 3c,d, where the hysteresis loops are shifted for clarity. The hysteresis indicates possible ferromagnetic components existing in ASb_3_Mn_9_O_19_, which persists until 40 (45) K for KSb_3_Mn_9_O_19_ (RbSb_3_Mn_9_O_19_). The coercive field of KSb_3_Mn_9_O_19_ (RbSb_3_Mn_9_O_19_) at 1.8 K is ≈0.34 T (0.06 T) while the remanent moment is ≈0.22 *µ*_B_/f.u. (≈0.14 *µ*_B_/f.u.). A slight difference of the hysteresis loops can be observed in ASb_3_Mn_9_O_19_. When the direction of the applied magnetic field is inverted, the magnetization of RbSb_3_Mn_9_O_19_ displays a sudden drop/increase, while KSb_3_Mn_9_O_19_ does not. The observation of the hysteresis indicates the possibilities of static spin freezing in ASb_3_Mn_9_O_19_. However, such spin freezing does not exist in the long‐range, as discussed later in Sections 2.7, 2.8.

### 2.5 Single Valency and Oxidation State of Mn

From the CW fitting results, the fitted *µ*_eff_ suggests the Mn^3+^ oxidation state in KSb_3_Mn_9_O_19_ since it is close to the spin‐only moment of trivalent Mn. However, the fitted *µ*_eff_ of RbSb_3_Mn_9_O_19_ exhibits a higher value (≈5.51 *µ*_B_/Mn), which can be attributed to two possible reasons: there are unaccounted contributions from orbital angular momentum, as described in Section 2.4; there is an additional oxidation state, e.g., Mn^2+^, which can result in a larger number of unpaired electrons as well as a bigger *µ*_eff_. In order to confirm the number of valencies and the oxidation states of Mn, XPS measurements were performed on both samples for the binding energy region of Mn 2*p* orbitals. As shown in Figure S3a,b (Supporting Information), an excellent match between the observed curve and fitted curve can be seen. Shirley background and Gaussian peak shape were applied to the fittings. For both compounds, a single pair of 2*p*_3/2_ and 2*p*_1/2_ peaks are found, along with a pair of satellite peaks at slightly higher binding energy, as observed in other Mn‐based systems,^[^ ^74^, ^75^ ^]^ implying single valency for Mn in ASb_3_Mn_9_O_19_. The binding energy of 2*p*_3/2_ and 2*p*_1/2_ peaks in KSb_3_Mn_9_O_19_ are 640.61 and 652.50 eV, while for RbSb_3_Mn_9_O_19_ they are 640.59 and 652.43 eV. The similarity of binding energies indicates that Mn in both compounds possesses a similar oxidation state, i.e., Mn^3+^ (*S* = 2). To further prove this, we performed syntheses of ASb_3_Mn_9_O_19_ in air by using Mn_2_O_3_ as starting material, instead of MnO. The powder XRD pattern with Rietveld fitting of KSb_3_Mn_9_O_19_ is shown in Figure S4 (Supporting Information), which exhibits high purity and a great match between the observed crystal structure and the observed powder XRD pattern. Considering that Mn_2_O_3_ is not likely to be reduced in air under high temperatures, this further proves that Mn in ASb_3_Mn_9_O_19_ should adopt oxidation states ≥ 3+. Therefore, the larger fitted ɛ_eff_ should originate from unaccounted orbital angular momentum contributions.

### 2.6 Heat Capacity

**Figure 5:**
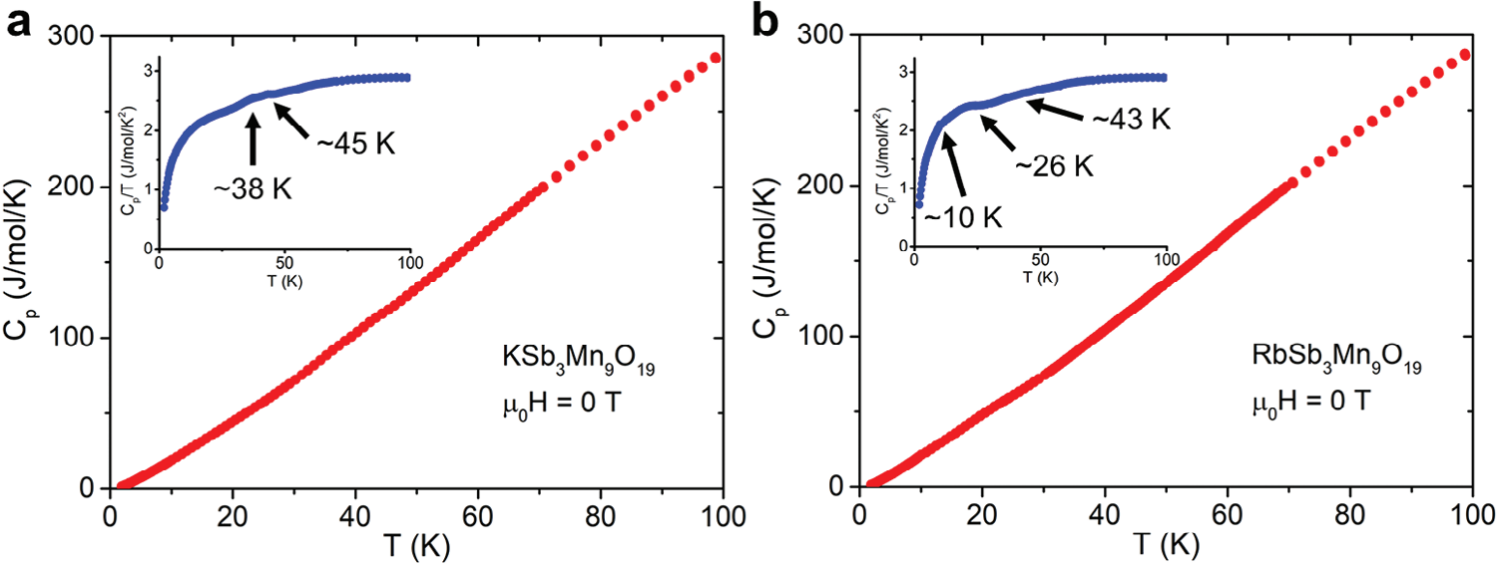
Temperature‐dependence of heat capacity (Cp) between 2 and 100 K under zero magnetic field of a) KSb_3_Mn_9_O_19_ and b) RbSb_3_Mn_9_O_19_. The insets show the temperature dependence of Cp/T.

To investigate whether long‐range magnetic order exists in ASb_3_Mn_9_O_19_, temperature‐dependent heat capacity (C_p_) measurements were conducted under zero magnetic field from 2 to 100 K. As shown in the main panels of **Figure** **5**a,b, (Supporting Information), C_p_ of both materials decreases monotonically and approximately linearly with decreasing temperature. Two small kinks can be seen in the inset of Figure 5a for KSb_3_Mn_9_O_19_ at ≈38 and ≈45 K, which shows the temperature‐dependent C_p_/T. The two kinks are likely to be relevant to T_3_ and T_4_ illustrated in Figure 3c. Kinks at ≈10, ≈26 and ≈43 K can also be observed in the C_p_/T versus T curve of RbSb_3_Mn_9_O_19_, likely correlated with T_1_, T_2,_ and T_3_ in Figure 3d. Additionally, no obvious peak/λ‐anomaly is seen in the heat capacity of both compounds, suggesting that the associated magnetic entropy is minimal. The typical reasons for a small magnetic entropy associated with a long‐range magnetic ordering include 1. The magnetic moments of the species are small; 2. The fraction of magnetic species that participates in the magnetic ordering is low. Given the Mn^3+^ (*S* = 2) from magnetic susceptibility and XPS results, which correspond to a large magnetic moment, we speculate that the reason for the minimal heat capacity peaks is that the fraction of Mn participating in the magnetic ordering in ASb_3_Mn_9_O_19_ is limited. This is qualitatively consistent with the short‐range magnetic order indicated by the neutron diffraction data of RbSb_3_Mn_9_O_19_ below.

### 2.7 Incommensurate Magnetic Order, 2D Magnetic Correlation and Magnetic Frustration

**Figure 6:**
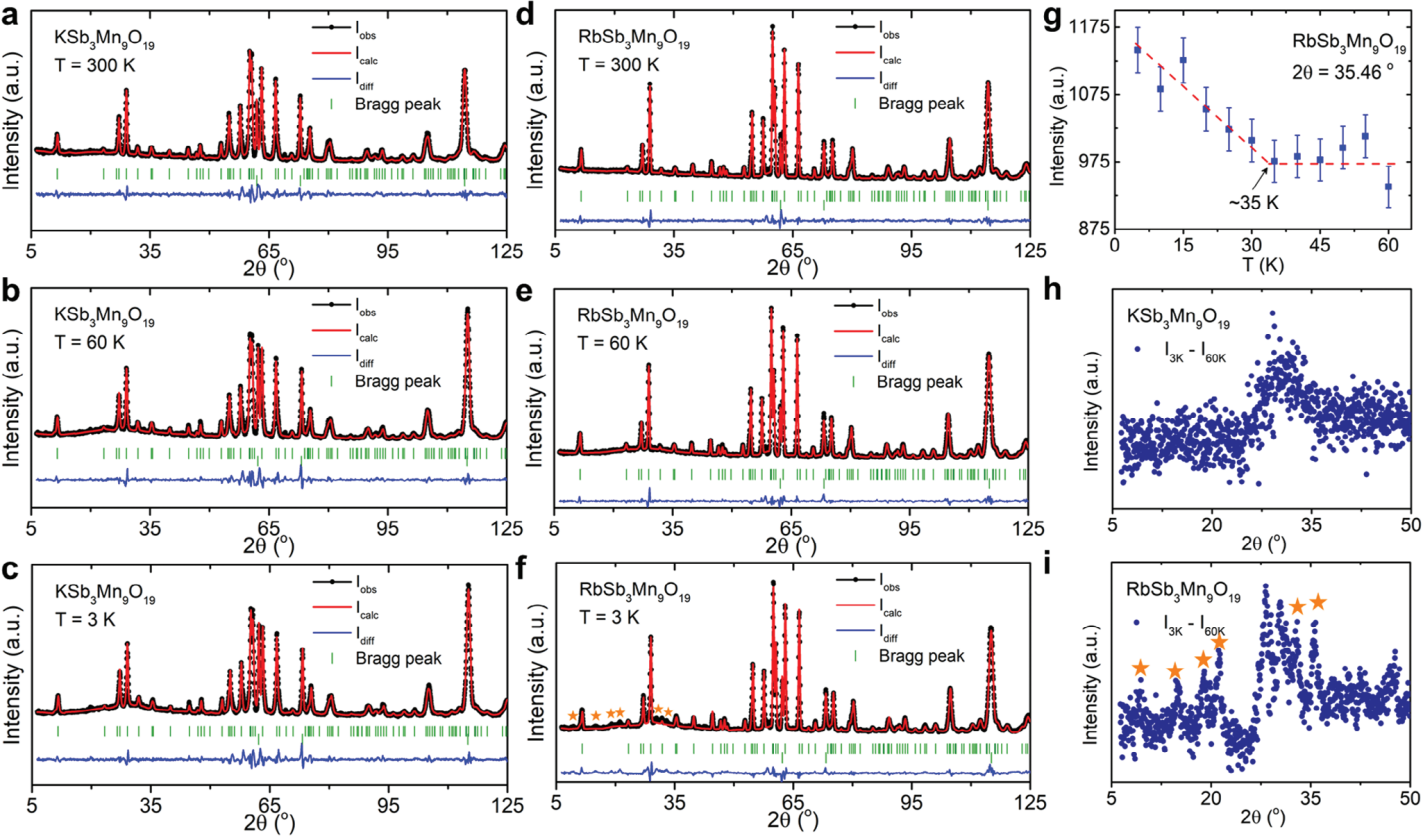
a–f) Neutron powder diffraction patterns of ASb_3_Mn_9_O_19_ under 300, 60, and 3 K with Rietveld fitting. The black solid circle with the line and red line represent the observed and calculated patterns, respectively. The blue line stands for the difference between the observed and calculated intensities. The green vertical bars are the Bragg peak positions for ASb_3_Mn_9_O_19_ (top) and the aluminum can for neutron powder diffraction (bottom). g) The evolution of peak intensity at 35.46o for RbSb_3_Mn_9_O_19_. h. and i) The temperature subtraction results of both ASb_3_Mn_9_O_19_ where I3–I60 K is shown.

To better interpret the magnetic behavior and heat capacity of ASb_3_Mn_9_O_19_, NPD was performed on ASb_3_Mn_9_O_19_ powder directly from the reaction crucibles at Oak Ridge National Laboratory. **Figure** **6**a–f show the NPD patterns of ASb_3_Mn_9_O_19_ with Rietveld refinement measured under 300, 60, and 3 K. No additional magnetic peaks are observed when KSb_3_Mn_9_O_19_ is cooled to either 60 or 3 K, indicating the absence of long‐range magnetic order. However, at least six magnetic peaks emerge at 3 K for RbSb_3_Mn_9_O_19_ below 35°. These sharper magnetic peaks sit on top of broad diffuse scattering, suggesting that not all the Mn sites give rise to these peaks. Meanwhile, the width of the magnetic peaks is larger than that of nuclear peaks, as shown in Figure S5 (Supporting Information) by comparing the full width at half maximum (FWHM) of the nuclear and magnetic peaks, revealing that RbSb_3_Mn_9_O_19_ is quasi‐long‐range ordered. It is noteworthy that the new peaks in RbSb_3_Mn_9_O_19_ only exist at low Q/2θ, matching the magnetic form factor, while the nuclear structure refinements at 3 and 60 K do not reveal nuclear structural transition. Therefore, the new peaks indeed originate from the symmetry change of the magnetic structure.

The plot shown in Figure 6g implies a monotonic increase of peak intensity, not the peak area, at 2θ = 35.46° below ≈35 K, implying that quasi‐long‐range magnetic order corresponding to the magnetic peaks in RbSb_3_Mn_9_O_19_ onsets at ≈35 K. The Bragg peak position is selected due to the relatively weaker diffuse scattering at this 2θ such that an obvious increasing intensity can be observed with temperature. Considering that the NPD was measured under zero magnetic field, the onset of quasi‐long‐range magnetic order in RbSb_3_Mn_9_O_19_ should correspond to T_2_ shown in Figure 3d. Efforts were made to index the magnetic propagation vector (*k*‐vector) for the magnetic peaks. However, no commensurate *k*‐vectors could be found. This implies that the magnetic structure of the quasi‐long‐range magnetic ordered phase in RbSb_3_Mn_9_O_19_ likely possesses an incommensurate magnetic structure. Furthermore, searches of incommensurate *k‐*vectors along high‐symmetry lines of the Brillouin zone also returned no feasible solutions, suggesting that the incommensurate *k*‐vector is of low symmetry. Given that there are also three distinct Mn sites, it is very difficult to figure out the incommensurate *k*‐vector with just the powder diffraction patterns.

Other than the incommensurate magnetic order in RbSb_3_Mn_9_O_19_, short‐range spin order exists in both KSb_3_Mn_9_O_19_ and RbSb_3_Mn_9_O_19_. Figure 6h,i exhibit the subtraction of NPD patterns of 300 K from that of 3 K (I_3K_ – I_60K_), while Figure S6 (Supporting Information) presents I_3K_ – I_300K_ and I_60K_ – I_300K_ to show the evolution of the NPD patterns. The minor data points scattered vertically from the majority are due to the thermal expansion of the nuclear peaks, while the magnetic peaks in RbSb_3_Mn_9_O_19_ are marked with stars. Note that the subtraction of the high‐temperature data from the low‐temperature one removes the temperature‐independent instrument background, and the resulting patterns are intrinsic to ASb_3_Mn_9_O_19_. Interestingly, strong diffuse scattering can be seen in both compounds, indicated by the broad peak centered at ≈29°. The broad peak indicates that spin–spin correlations are local rather than long‐range. The strong magnetic diffuse scattering observed in NPD usually originates from 2D short‐range magnetic correlations. Similar behavior was reported in 2D short‐range ordered frustrated spinels, such as Li_2_Mn_2_O_4_.^[^ ^76^, ^77^, ^78^ ^]^ Considering the lack of λ‐anomaly in heat capacity measurements, as well as the puckered honeycomb, Kagome, and triangular sublattices of Mn in ASb_3_Mn_9_O_19_, it is likely that magnetic frustration exists in ASb_3_Mn_9_O_19_. If taking T_4_ in Figure 3c,d as the first magnetic anomaly temperature, the frustration factors are 3.4 and 3.8 for KSb_3_Mn_9_O_19_ and RbSb_3_Mn_9_O_19_, respectively. The synthesis of single crystals is indeed needed for further investigations on the magnetism in ASb_3_Mn_9_O_19_.

### 2.8 Reverse Monte Carlo Refinement

**Figure 7:**
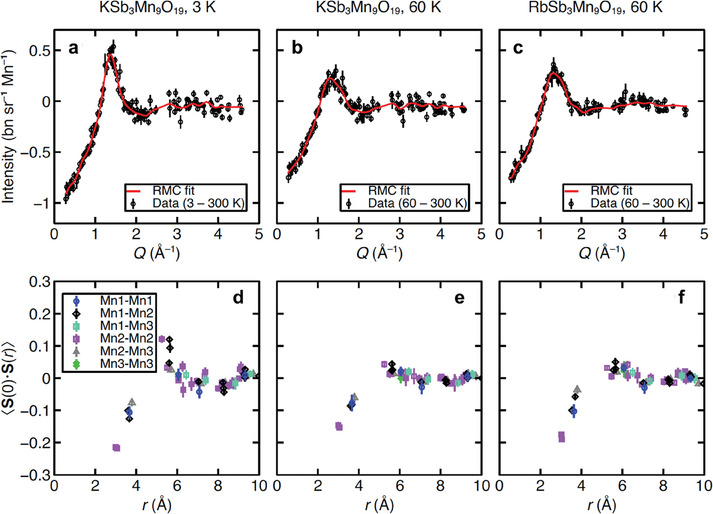
Magnetic diffuse scattering in the paramagnetic phase fitted by the Spinvert program of a) KSb_3_Mn_9_O_19_ at 3 K, b) KSb_3_Mn_9_O_19_ at 60 K, and c) RbSb_3_Mn_9_O_19_ at 60 K. d–f) display the radial dependence of the spin‐pair correlation function. The signs indicate that the spins are parallel (+) or antiparallel (‐). The values stand for the collinearity between spins.

To determine the nature of the short‐range spin correlations, reverse Monte Carlo (RMC) refinement as implemented in the Spinvert program^[^ ^79^ ^]^ was employed to fit the magnetic diffuse scattering. Details of the data processing and refinement parameters are given in Experimental Details. **Figure** **7**a–c show the experimental diffuse scattering data with 300 K data subtracted, and the RMC fits for KSb_3_Mn_9_O_19_ at 3 K, KSb_3_Mn_9_O_19_ at 60 K, and RbSb_3_Mn_9_O_19_ at 60 K, respectively (we note the data for RbSb_3_Mn_9_O_19_ at 3 K could not be modeled using this approach due to the presence of sharp incommensurate peaks). At 60 K, the diffuse scattering data is similar for A = K and Rb. At 3 K, the diffuse scattering data for A = K shows sharper features compared to 60 K, indicating an increase in the magnetic correlation length on cooling the sample. To quantify the magnetic correlations, the spin‐spin correlation function <**S**(0).**S**(r)> was calculated and is shown in Figure 7d–f for KSb_3_Mn_9_O_19_ at 3 K, KSb_3_Mn_9_O_19_ at 60 K, and RbSb_3_Mn_9_O_19_ at 60 K, respectively. This quantity is equivalent to the magnetic pair distribution function for magnetically‐isotropic systems; it is equal to +1 if spin pairs separated by distance *r* are perfectly aligned on average, and to –1 if perfectly anti‐aligned. It reveals that antiferromagnetic correlations persist at 60 K for both A = K and Rb, with similar magnitudes in both compounds. The strongest correlation is antiferromagnetic and occurs in a short‐range manner (⪅ 4 Å) between Mn2–Mn2, i.e., within the Kagome layers, which is consistent with its shortest Mn–Mn distance. Other than Mn2–Mn2, weaker magnetic correlation can be observed between Mn1–Mn1, Mn1‐Mn2, and Mn2‐Mn3 pairs;, i.e., primarily within Kagome‐honeycomb bilayers, suggesting mainly 2D correlations. Given that similar magnetic diffuse scattering was observed in other 2D short‐range ordered frustrated magnets,^[^ ^76^, ^77^, ^78^ ^]^ we can conclude that 2D magnetic correlation indeed exists in ASb_3_Mn_9_O_19_. At 3 K for A = Rb, the spin correlations show similar trends to at 60 K, but with larger near‐neighbor antiferromagnetic correlations and smaller but significant ferromagnetic correlations at ≈5.24 Å. The Spinvert fitting confirms the lack of long‐range magnetic order in KSb_3_Mn_9_O_19_ down to a temperature of 3 K, and the existence of antiferromagnetic short‐range order in both compounds at 60 K. Together with the values of the frustration parameter mentioned in Section 2.7, this suggests appreciable magnetic frustration.

### 2.9 Electrical Resistivity

**Figure 8:**
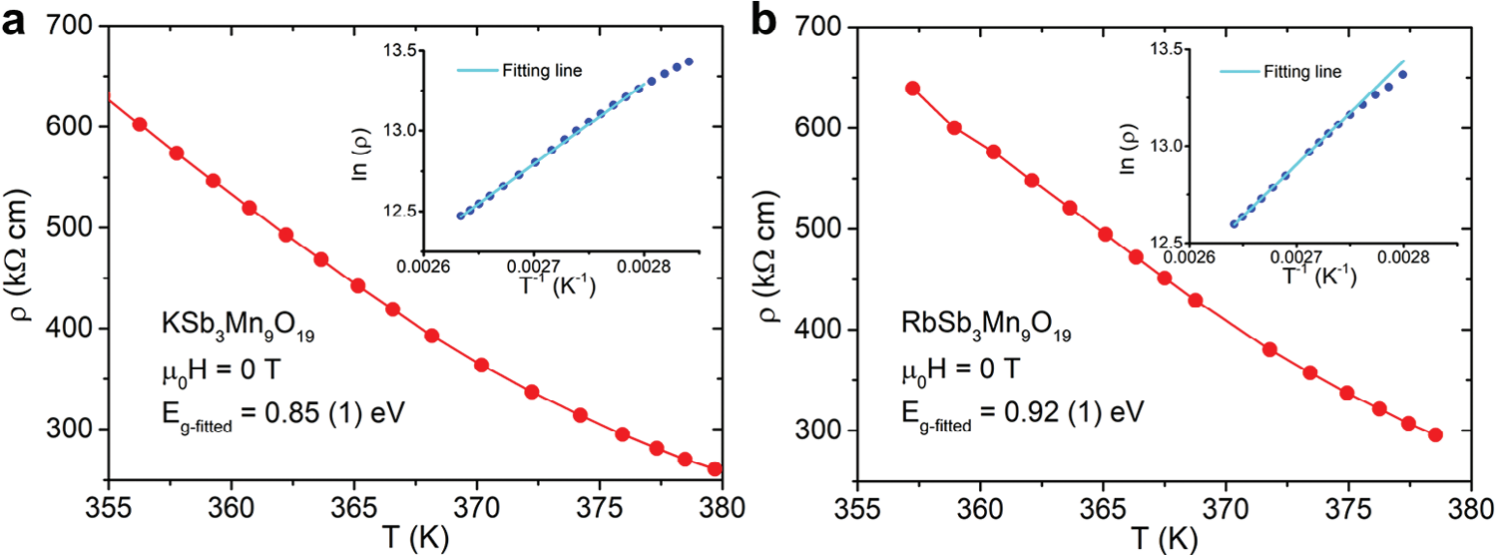
Temperature‐dependent electrical resistivity of a). KSb_3_Mn_9_O_19_ and b). RbSb_3_Mn_9_O_19_ from ≈355 to ≈380 K under no external magnetic field. The insets show the Arrhenius fitting on the lnρ vs T‐1 plots.

Temperature‐dependent electrical resistivity of ASb_3_Mn_9_O_19_ under zero magnetic field were illustrated in **Figure** **8**a,b. Both materials exhibit a similar trend of temperature dependence where the resistivity increases monotonically with decreasing temperature, indicating a semiconducting/insulating nature. The signal is out of the instrument's measurable range below the lowest measured temperatures (⪅ 350 K). To obtain the electronic bandgap (*E_g_*), the Arrhenius equation

(2) $$\rho \;=\rho_{0}\;e^{E_{g}}{/}_{2k_{B}T}$$

where ρ_0_ is the pre‐exponential term and is a constant, and *k_B_* is Boltzmann's constant, was employed to fit the data, as shown in the insets of Figure 8a,b. The fitting was applied only to the highest temperatures where a linear relation between *ln*ρ and 1/T can be found. The fitted bandgap for KSb_3_Mn_9_O_19_ (*E*_*g* − *K*_) is 0.85 (1) eV and *E*_*g* − *Rb*_ is 0.92 (1) eV, indicating that both KSb_3_Mn_9_O_19_ and RbSb_3_Mn_9_O_19_ are semiconductors.

## 3 Conclusion

Herein, we present the discovery, synthesis, and characterization of magnetic properties of the first Mn‐based magnetoplumbite phase (*M*‐type hexaferrites), ASb_3_Mn_9_O_19_ (A = K or Rb). Single‐phase polycrystalline samples were synthesized using the high‐temperature solid‐state method. Single‐crystal XRD revealed a crystal structure of both compounds, isostructural to magnetoplumbites. DC magnetic susceptibility and magnetization indicated strong antiferromagnetic coupling under high temperatures and Mn^3+^ (*S* = 2) for ASb_3_Mn_9_O_19_ as well as multiple magnetic features under low temperatures. However, the temperature‐dependent heat capacity did not exhibit any prominent λ‐anomaly. After performing NPD, we concluded that KSb_3_Mn_9_O_19_ is not long‐range magnetically ordered within the measured temperature range, while an incommensurate magnetic structure can be found in RbSb_3_Mn_9_O_19_. Moreover, both compounds show short‐range magnetic order, potentially originating from magnetic frustration, as evidenced by strong diffuse scattering in the NPD data and the absence of prominent λ‐anomaly in the heat capacity. Therefore, the discovery of the first Mn‐based magnetoplumbite phase provides a promising platform for studying the intertwining of different geometrically frustrated sublattices of magnetic species. Further investigations on ASb_3_Mn_9_O_19_, especially on single crystal samples, are necessary to uncover their detailed magnetic structure and identify the origin of magnetic frustration. Additionally, the newly discovered Mn‐based magnetoplumbites with *S* = 2 provide an excellent playground for investigating integer‐spin‐frustrated magnets.

## 4 Experimental Section

### Synthesis of Polycrystalline ASb_3_Mn_9_O_19_

Polycrystalline ASb_3_Mn_9_O_19_ (A = K or Rb) was prepared using a high‐temperature solid‐state method. Stoichiometric mixture of anhydrous K_2_CO_3_ (99%, Thermo Scientific), Rb_2_CO_3_ (99.8%, Thermo Scientific), Sb_2_O_3_ (99.9%, Thermo Scientific), and MnO (99.99%, Thermo Scientific) with 60% and 40% excess of K_2_CO_3_ and Rb_2_CO_3_, respectively, were thoroughly ground and placed in alumina crucibles. The excess amount of A_2_CO_3_ was used to compensate for their vaporization under high temperatures. The mixture was subsequently heated to 1200 °C and kept for 12 h followed by air quenching. Both samples were re‐annealed at the same temperature overnight several times with intermediate grindings until pure phases were obtained. The total heating time under 1200 °C needed for single‐phase products was usually less than 36 h. The obtained products were black powder and air‐stable. Small hexagonal crystals (up to ≈100×100×10 *µ*m^3^) could be found within all batches. Polycrystalline KSb_3_Mn_9_O_19_ could also be synthesized by replacing MnO with Mn_2_O_3_ (98%, Thermo Scientific), which exhibited the same results in powder and single crystal X‐ray diffraction measurements. However, synthesis of polycrystalline RbSb_3_Mn_9_O_19_ using Mn_2_O_3_ only yielded minor target compounds along with impurities such as Mn_3_O_4_ and Mn_2_Sb_2_O_7_. The failure of synthesis might be due to the higher reactivity of Rb_2_CO_3_ as well as the lower reactivity of Mn_2_O_3_ such that Rb_2_CO_3_ vaporized before the reaction could start or Mn_2_O_3_ was oxidized prior to the reaction temperature. Therefore, in this paper, all the samples used for properties characterizations were made from MnO.

### Single Crystal and Powder XRD

Multiple crystals (≈50×50×10 *µ*m^3^) of both KSb_3_Mn_9_O_19_ and RbSb_3_Mn_9_O_19_ were picked for single crystal XRD for crystal structure and chemical composition determination. A Bruker D8 QUEST ECO diffractometer equipped with APEX4 software and Mo radiation (λ_Kα _= 0.71073 Å) was employed for single crystal XRD measurements at room temperature. Crystals were soaked in glycerol and then mounted using a Kapton loop. The Bruker SMART software was utilized for data acquisition while the corrections for Lorentz and polarization effects were included. Numerical absorption correction was made through a crystal‐face‐indexing method using *XPREP*. The direct method and full‐matrix least‐squares on F^2^ procedure within the SHELXTL package were employed to solve the crystal structure.^[^ ^80^, ^81^ ^]^

Powder XRD measurements were utilized to determine the phase purity for the polycrystalline samples. A Bruker D2 PHASER with Cu Kα radiation and a LynxEye‐XE detector was employed. The resulting powder XRD patterns were fitted by the Rietveld method using Fullprof Suite.^[^ ^82^ ^]^ Crystal structures determined by single crystal XRD were used to obtain the calculated powder XRD patterns and to fit observed patterns.

### Physical Property Measurement

The DC magnetic susceptibility (χ), defined as χ = M/H where M is the observed magnetic moment and H is the applied magnetic field, was measured in a Quantum Design physical property measurement system (PPMS) Dynacool (1.8–300 K, 0–9 T) equipped with an ACMS II function from 2 to 300 K under various applied magnetic fields. Field‐dependent magnetization data was collected at multiple temperatures with applied magnetic fields ranging from −9 to 9 T. The resistivity measurements were carried out in the PPMS using the four‐probe method between 1.8 to 300 K. Platinum wires were attached to the samples by silver epoxy to ensure ohmic contact. Heat capacity was measured using a standard relaxation method in the PPMS, with a ^3^He function for data below 1.8 K. All the physical properties measurements were conducted on powder samples (for magnetic measurements) or annealed pelletized samples (for resistivity and heat capacity measurements). For the annealed pelletized samples, they were pressed under a hydraulic press at ≈20 MPa for 5 min before being annealed under 1200 °C for 10 h. The resulting annealed pellets were utilized for resistivity and heat capacity measurements.

### Scanning Electron Microscopy with Energy‐Dispersive Spectroscopy (SEM‐EDS)

Compositional analysis was performed via scanning electron microscopy (SEM) with energy‐dispersive spectroscopy (EDS). A Zeiss Sigma 500 VP SEM with Oxford Aztec X‐EDS was used with an electron beam energy of 20 kV.

### XPS

XPS was performed in a Thermo Scientific K‐AlphaPlus instrument equipped with monochromatic Al K_α_ radiation (1486.7 eV) as the excitation source. The X‐ray analysis area for measurement was set at 200 × 400 *µ*m (ellipse shape) and a flood gun was used for charge compensation. The pass energy was 200 eV for the wide (survey) spectra and 50 eV for the high‐resolution regions (narrow spectra). The base pressure of the analysis chamber was less than ≈1 × 10^−9^ mbar. The analysis chamber pressure was at 1 × 10^−7^ mbar during data acquisition. Data were collected and processed using the Thermo Scientific Avantage XPS software package. Pelletized polycrystalline samples used for XPS measurements were treated the same way as samples used for resistivity and heat capacity measurements.

### NPD

NPD measurements were carried out on the HB‐2A powder diffractometer at the High Flux Isotope Reactor (HFIR), Oak Ridge National Laboratory (ORNL).^[^ ^83^, ^84^ ^]^ Constant wavelength measurements were performed at 2.41 Å from the Ge(113) monochromator reflection. A pyrolytic graphite filter was placed before the sample to remove higher order reflections. The pre‐mono, pre‐sample and pre‐detector collimation was open–open‐12′. The samples were contained in a 6 mm diameter aluminum can and cooled in a closed‐cycle refrigerator in the temperature range 3–300 K. The diffraction pattern was collected by scanning a 120° bank of 44 3He detectors in 0.05° steps to give 2Θ coverage from 5° to 130°.

### Reverse Monte Carlo Refinements

Neutron powder diffraction data were first placed in absolute intensity units (bn/sr/Mn) by normalization to the nuclear Bragg profile. To remove the background and isolate the magnetic scattering signal, data collected at 300 K were subtracted from the data collected at lower temperatures (3 or 60 K), and data points were excluded where the calculated nuclear Bragg signal exceeded a threshold value. Data were binned in intervals of 0.02 Å^−1^. The magnetic scattering obtained in this way was used as input data for reverse Monte Carlo refinements using the Spinvert program.^[^ ^79^ ^]^ Refinements with supercells of 8×8×2 crystallographic unit cells (2304 Mn spins). It was assumed that all spins are *S* = 2 with magnetic moments of 4.90 *µ*_B_ per Mn, and the Mn1, Mn2, and Mn3 sites were fully occupied; possible Mn occupancy on the Sb1 site was neglected. Refinements were performed for 100 proposed rotations per spin, and a flat background level was allowed to refine to account for changes in the background level between 60 and 300 K. Good agreement with the data was obtained with these parameters.

CCDC 2403656–2403657 contains the supplementary crystallographic data for this paper. These data can be obtained free of charge from The Cambridge Crystallographic Data Centre via www.ccdc.cam.ac.uk/data_request/cif.

## Conflict of Interest

The authors declare no conflict of interest.

## Supporting information



Supporting Information

## Data Availability

The data that support the findings of this study are available from the corresponding author upon reasonable request.
