# Supplementary material for: ASb3Mn9O19 (A = K or Rb): New Mn‐Based 2D Magnetoplumbites with Geometric and Magnetic Frustration
Source: Adv Mater. 2024 Dec 31;37(10):2417906. doi: 10.1002/adma.202417906 (PMC11899497; doi:10.1002/adma.202417906)
Supplement: Supplementary file 1 — Supporting Information [file ADMA-37-2417906-s001.docx]

***Supporting Information***

**ASb_3_Mn_9_O_19_ (A = K or Rb): New Mn-Based Two-Dimensional Magnetoplumbites with Geometric and Magnetic Frustration**

*Jianyi Chen*, *Stuart Calder, Joseph A. M. Paddison, Gina Angelo, Liana Klivansky, Jian Zhang, Huibo Cao* *and Xin Gui**

Jianyi Chen, Gina Angelo, Xin Gui

Department of Chemistry, University of Pittsburgh, Pittsburgh, PA, 15260, USA

E-mail: [xig75@pitt.edu](mailto:xig75@pitt.edu)

Stuart Calder, Joseph A. M. Paddison, Huibo Cao

Neutron Scattering Division, Oak Ridge National Laboratory, Oak Ridge, TN, 37831, USA

Liana Klivansky, Jian Zhang

The Molecular Foundry, Lawrence Berkeley National Laboratory, Berkeley, CA, 94720, USA

**Table of Contents**

[Table S1](#_Toc496175023)  2

[Table S2](#_Toc496175023) 3

[Table S3](#_Toc496175023) 4

[Table S4](#_Toc496175023) 5

[Table S5](#_Toc496175023) 6

[Table S6](#_Toc496175023) 7

[Table S7](#_Toc496175023) 8

[Figure S1](#_Toc496175023) 9

[Figure S2](#_Toc496175023) 10

[Figure S3](#_Toc496175023) 11

[Figure S4](#_Toc496175023) 12

[Figure S5](#_Toc496175023) 13

[Figure S6](#_Toc496175023) 14

**Table S1**. Atomic coordinates and equivalent isotropic displacement parameters for ASb_3_Mn_9_O_19_ (A = K or Rb) at 293 (2) K. (U*_eq_* is defined as one-third of the trace of the orthogonalized U*_ij_* tensor (Å^2^))

KSb_2.82(1)_Mn_9.18(1)_O_19_:

| Atom | Wyck. | | Occ. | *x* | *y* | *Z* | *U_eq_* |
| --- | --- | --- | --- | --- | --- | --- | --- |
| Sb1 | 4*f* | 1 | | 1/3 | 2/3 | 0.18971 (2) | 0.0100 (1) |
| Sb2 | 2*a* | 0.82 (1) | | 0 | 0 | 0 | 0.0079 (3) |
| Mn4 | 2*a* | 0.18 (1) | | 0 | 0 | 0 | 0.0079 (3) |
| Mn1 | 4*f* | 1 | | 1/3 | 2/3 | 0.02130 (6) | 0.0135 (3) |
| Mn2 | 12*k* | 1 | | 0.83341 (8) | 0.16659 (8) | 0.10877 (3) | 0.0105 (2) |
| Mn3 | 2*b* | 1 | | 0 | 0 | ¼ | 0.0201 (5) |
| K1 | 2*d* | 1 | | 2/3 | 1/3 | ¼ | 0.0181 (5) |
| O1 | 12*k* | 1 | | 0.1488 (4) | 0.8512 (4) | 0.0508 (2) | 0.0181 (8) |
| O2 | 12*k* | 1 | | 0.4949 (5) | 0.5051 (5) | 0.1508 (2) | 0.0149 (7) |
| O3 | 6*h* | 1 | | 0.1943 (5) | 0.3885 (11) | ¼ | 0.012 (1) |
| O4 | 4*f* | 1 | | 2/3 | 1/3 | 0.0670 (3) | 0.020 (2) |
| O5 | 4*e* | 1 | | 0 | 0 | 0.1480 (3) | 0.025 (2) |

RbSb_2.93(1)_Mn_9_O_19_:

| Atom | Wyck. | | Occ. | *x* | *y* | *z* | *U_eq_* |  |
| --- | --- | --- | --- | --- | --- | --- | --- | --- |
| Sb1 | 4*f* | 1 | | 1/3 | 2/3 | 0.18960 (2) | 0.0075 (1) |  |
| Sb2 | 2*a* | 0.931 (5) | | 0 | 0 | 0 | 0.0065 (2) |  |
| Mn1 | 4*f* | 1 | | 1/3 | 2/3 | 0.02146 (6) | 0.0125 (3) |  |
| Mn2 | 12*k* | 1 | | 0.83332 (8) | 0.16668 (8) | 0.10868 (3) | 0.0089 (2) |  |
| Mn3 | 2*b* | 1 | | 0 | 0 | ¼ | 0.0196 (4) |  |
| Rb1 | 2*d* | 1 | | 2/3 | 1/3 | ¼ | 0.0124 (2) |  |
| O1 | 12*k* | 1 | | 0.1485 (4) | 0.8515 (4) | 0.0503 (2) | 0.0170 (7) |  |
| O2 | 12*k* | 1 | | 0.4941 (4) | 0.5059 (4) | 0.1503 (1) | 0.0114 (6) |  |
| O3 | 6*h* | 1 | | 0.1940 (5) | 0.3879 (10) | ¼ | 0.0091 (8) |  |
| O4 | 4*f* | 1 | | 2/3 | 1/3 | 0.0665 (3) | 0.017 (1) |  |
| O4 | | 4*e* | 1 | | 0 | 0 | 0.1477 (3) | 0.029 (2) |

**Table S2.** Anisotropic thermal displacement parameters for ASb_3_Mn_9_O_19_ (A = K or Rb) at 293 (2) K.

KSb_2.82(1)_Mn_9.18(1)_O_19_:

| Atom | | U11 | U22 | U33 | U12 | U13 | U23 |
| --- | --- | --- | --- | --- | --- | --- | --- |
| Sb1 | | 0.0117 (2) | 0.0117 (2) | 0.0066 (2) | 0.0058 (1) | 0 | 0 |
| Sb2 | | 0.0091 (3) | 0.0091 (3) | 0.0054 (4) | 0.0046 (2) | 0 | 0 |
| Mn4 | | 0.0091 (3) | 0.0091 (3) | 0.0053 (4) | 0.0046 (2) | 0 | 0 |
| Mn1 | | 0.0139 (4) | 0.0139 (4) | 0.0127 (6) | 0.0069 (2) | 0 | 0 |
| Mn2 | 0.0115 (3) | | 0.0115 (3) | 0.0105 (3) | 0.0074 (3) | -0.0001 (1) | 0.0001 (1) |
| Mn3 | 0.0099 (5) | | 0.0099 (5) | 0.0404 (13) | 0.0050 (3) | 0 | 0 |
| K1 | 0.0188 (8) | | 0.0188 (8) | 0.017 (1) | 0.0094 (4) | 0 | 0 |

RbSb_2.93(1)_Mn_9_O_19_:

| Atom | U11 | U22 | U33 | U12 | U13 | U23 |
| --- | --- | --- | --- | --- | --- | --- |
| Sb1 | 0.0084 (2) | 0.0084 (2) | 0.0057 (2) | 0.0042 (1) | 0 | 0 |
| Sb2 | 0.0070 (3) | 0.0070 (3) | 0.0054 (3) | 0.0035 (1) | 0 | 0 |
| Mn1 | 0.0130 (4) | 0.0130 (4) | 0.0114 (5) | 0.0065 (2) | 0 | 0 |
| Mn2 | 0.0098 (2) | 0.0098 (2) | 0.0096 (3) | 0.0067 (3) | -0.0003 (1) | 0.0003 (1) |
| Mn3 | 0.0069 (5) | 0.0069 (5) | 0.0449 (13) | 0.0035 (3) | 0 | 0 |
| Rb1 | 0.0126 (3) | 0.0126 (3) | 0.0119 (4) | 0.0063 (2) | 0 | 0 |
| O5 | 0.038 (3) | 0.038 (3) | 0.010 (3) | 0.019 (2) | 0 | 0 |

**Table S3.** Comparison of single crystal structure refinement parameters for KSb_3_Mn_9_O_19_ with Sb2 site mixed with Mn atom and constrained to be 100% occupied.

| Refined Formula | KSb_2.82(1)_Mn_9.18(1)_O_19_ | KSb_3_Mn_9_O_19_ |
| --- | --- | --- |
| Temperature (K) | 293 (2) | 293 (2) |
| F.W. (g/mol) | 1190.45 | 1202.81 |
| Space group; Z | *P* 6_3_/mmc; 2 | *P* 6_3_/*mmc*; 2 |
| *a*(Å) | 6.0606 (1) | 6.0606 (1) |
| *c*(Å) | 23.853 (1) | 23.853 (1) |
| V (Å^3^) | 758.76 (5) | 758.76 (5) |
| θ range (º) | 3.416-33.191 | 3.416-33.191 |
| No. reflections; *R_int_*  No. independent reflections  No. parameters | 21526; 0.0631 | 21526; 0.0631 |
|  | 620 | 620 |
|  | 32 | 32 |
| *R_1_: ωR_2_* (*I*>2δ(*I*)) | 0.0304; 0.0657 | 0.0380; 0.0908 |
| Goodness of fit | 1.199 | 1.178 |
| Diffraction peak and hole (e^-^/ Å^3^) | 1.308; -1.268 | 1.422; -3.665 |

**Table S4.** Comparison of single crystal structure refinement parameters for RbSb_3_Mn_9_O_19_ with Sb2 site relaxed and constrained to be 100% occupied.

| Refined Formula | RbSb_2.93(1)_Mn_9_O_19_ | RbSb_3_Mn_9_O_19_ |
| --- | --- | --- |
| Temperature (K) | 293 (2) | 293 (2) |
| F.W. (g/mol) | 1240.66 | 1249.81 |
| Space group; Z | *P* 6_3_/mmc; 2 | *R* 3m; 3 |
| *a*(Å) | 6.0818 (7) | 6.0818 (7) |
| *c*(Å) | 23.905 (4) | 23.905 (4) |
| V (Å^3^) | 765.8 (2) | 765.8 (2) |
| θ range (º) | 3.409-34.343 | 3.409-34.343 |
| No. reflections; *R_int_*  No. independent reflections  No. parameters | 21395; 0.0401 | 21395; 0.0401 |
|  | 681 | 681 |
|  | 34 | 32 |
| *R_1_: ωR_2_* (*I*>2δ(*I*)) | 0.0291; 0.0594 | 0.0343; 0.0766 |
| Goodness of fit | 1.387 | 1.325 |
| Diffraction peak and hole (e^-^/ Å^3^) | 1.174; -0.892 | 1.315; -3.354 |

**Table S5.** EDS results for KSb_3_Mn_9_O_19_.

| **Spectrum #** | **K** | **Sb** | **Mn** | **Mn/Sb Ratio** |
| --- | --- | --- | --- | --- |
| **Spectrum 1** | 3.1(1) | 9.0(2) | 28.9(2) | 3.2(1) |
| **Spectrum 2** | 3.1(1) | 9.2(2) | 29.6(2) | 3.2(1) |
| **Spectrum 3** | 3.0(1) | 8.9(2) | 28.4(2) | 3.2(1) |
| **Spectrum 4** | 3.0(1) | 9.0(2) | 28.0(2) | 3.1(1) |
| **Spectrum 5** | 2.9(1) | 8.8(2) | 27.6(2) | 3.1(1) |
| **Spectrum 6** | 2.9(1) | 8.6(2) | 27.9(2) | 3.2(1) |
| **Spectrum 7** | 3.0(1) | 8.7(2) | 27.6(2) | 3.2(1) |
| **Spectrum 8** | 3.1(1) | 9.1(2) | 28.3(2) | 3.1(1) |
| **Spectrum 9** | 2.6(1) | 8.0(2) | 24.6(2) | 3.1(1) |
| **Spectrum 10** | 3.0(1) | 8.7(2) | 27.5(2) | 3.2(1) |
| **Spectrum 11** | 3.0(1) | 9.3(2) | 31.4(2) | 3.4(1) |
| **Spectrum 12** | 3.0(1) | 8.8(2) | 28.9(2) | 3.3(1) |
| **Spectrum 13** | 2.8(1) | 8.7(2) | 27.9(2) | 3.2(1) |
| **Spectrum 14** | 3.1(1) | 8.5(2) | 27.9(2) | 3.3(1) |
| **Spectrum 15** | 3.0(1) | 8.8(2) | 28.1(2) | 3.2(1) |
| **Spectrum 16** | 2.9(1) | 8.8(2) | 28.3(2) | 3.2(1) |
| **Spectrum 17** | 2.7(1) | 7.7(2) | 24.1(2) | 3.1(1) |
| **Spectrum 18** | 3.0(1) | 8.6(2) | 27.7(2) | 3.2(1) |
| **Spectrum 19** | 2.9(1) | 8.9(2) | 27.7(2) | 3.1(1) |
| **Spectrum 20** | 3.0(1) | 8.6(2) | 27.5(2) | 3.2(1) |
| **Spectrum 21** | 3.0(1) | 8.8(2) | 27.4(2) | 3.1(1) |
| **Spectrum 22** | 2.8(1) | 8.6(2) | 28.0(2) | 3.3(1) |
| **Spectrum 23** | 2.9(1) | 8.5(2) | 27.9(2) | 3.3(1) |
| **Spectrum 24** | 3.0(1) | 8.7(2) | 27.1(2) | 3.1(1) |
| **Average** | 2.95 (10) | 8.73 (19) | 27.85 (0.20) | 3.19 (9) |
| **Normalize to K** | 1.00 (3) | 2.96 (6) | 9.44 (7) |  |

**Table S6.** EDS results for RbSb_3_Mn_9_O_19_.

| **Spectrum Label** | **Rb** | **Sb** | **Mn** | **Mn/Sb Ratio** |
| --- | --- | --- | --- | --- |
| **Spectrum 1** | 3.0(2) | 8.6(2) | 25.1(2) | 2.9(1) |
| **Spectrum 2** | 3.1(2) | 8.7(2) | 25.3(2) | 2.9(1) |
| **Spectrum 3** | 2.7(2) | 10.6(2) | 32.3(2) | 3.0(1) |
| **Spectrum 4** | 3.1(2) | 9.0(2) | 27.1(2) | 3.0(1) |
| **Spectrum 5** | 3.1(2) | 9.0(2) | 26.8(2) | 3.0(1) |
| **Spectrum 6** | 3.1(2) | 9.0(2) | 26.9(2) | 3.0(1) |
| **Spectrum 7** | 3.0(2) | 9.0(2) | 26.9(2) | 3.0(1) |
| **Spectrum 8** | 3.0(2) | 10.5(2) | 32.3(2) | 3.1(1) |
| **Spectrum 9** | 3.1(2) | 10.6(2) | 32.7(2) | 3.1(1) |
| **Spectrum 10** | 2.9(2) | 10.8(2) | 32.7(2) | 3.0(1) |
| **Spectrum 11** | 3.0(2) | 10.6(2) | 32.4(2) | 3.1(1) |
| **Spectrum 12** | 3.0(2) | 9.2(2) | 26.9(2) | 2.9(1) |
| **Spectrum 13** | 3.1(2) | 9.1(2) | 27.4(2) | 3.0(1) |
| **Spectrum 14** | 2.9(2) | 9.1(2) | 27.3(2) | 3.0(1) |
| **Spectrum 15** | 3.0(2) | 9.1(2) | 27.4(2) | 3.0(1) |
| **Spectrum 16** | 3.1(2) | 9.2(2) | 27.0(2) | 2.9(1) |
| **Spectrum 17** | 3.0(2) | 9.1(2) | 27.6(2) | 3.0(1) |
| **Spectrum 18** | 3.0(2) | 9.0(2) | 27.6(2) | 3.1(1) |
| **Spectrum 19** | 3.0(2) | 9.6(2) | 29.3(2) | 3.1(1) |
| **Spectrum 20** | 3.2(2) | 9.5(2) | 27.9(2) | 2.9(1) |
| **Spectrum 21** | 3.1(2) | 9.4(2) | 28.0(2) | 3.0(1) |
| **Spectrum 22** | 2.7(2) | 9.7(2) | 29.1(2) | 3.0(1) |
| **Spectrum 23** | 3.0(2) | 10.4(2) | 30.8(2) | 2.9(1) |
| **Average** | 3.01 (34) | 9.51 (20) | 28.56 (19) | 3.00 (9) |
| **Normalize to Rb** | 1.0 (1) | 3.16 (7) | 9.49 (6) |  |

**Table S7.** Comparison of Mn/Sb ratio obtained from single crystal X-ray diffraction (SCXRD) and energy-dispersive spectroscopy (EDS).

| **Sample name** | **Situation on Sb2 site** | **SCXRD** | **EDS** |
| --- | --- | --- | --- |
| **KSb_3_Mn_9_O_19_** | Mn-Sb mix | 3.26 (2) | 3.19 (9) |
|  | Sb with vacancy | 3.10 (1) |  |
| **KSb_3_Mn_9_O_19_** | Mn-Sb mix | 3.18 (2) | 3.00 (9) |
|  | Sb with vacancy | 3.07 (1) |  |


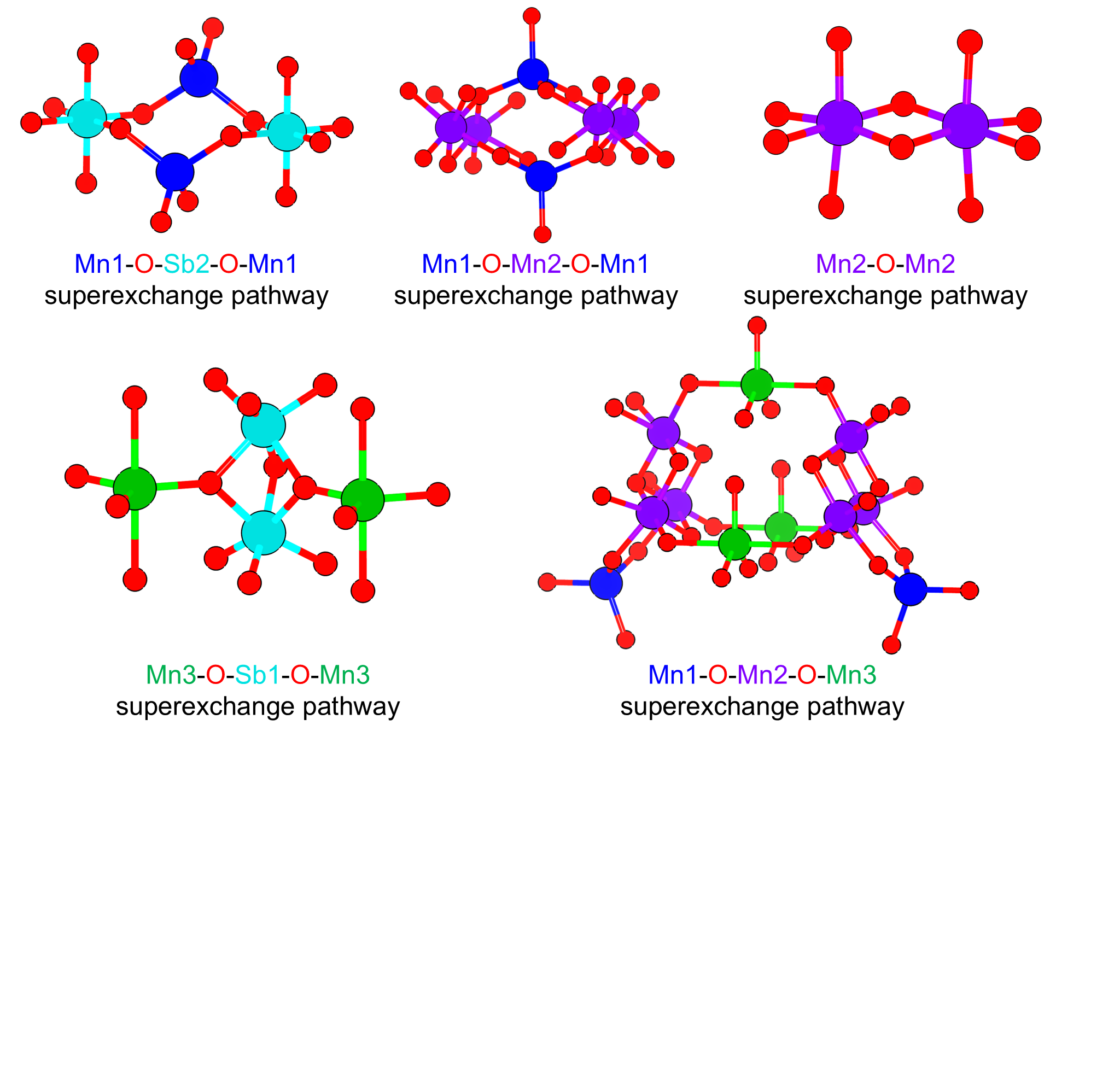
**Figure S1.** Possible Mn-Mn superexchange pathways in ASb_3_Mn_9_O_19_.


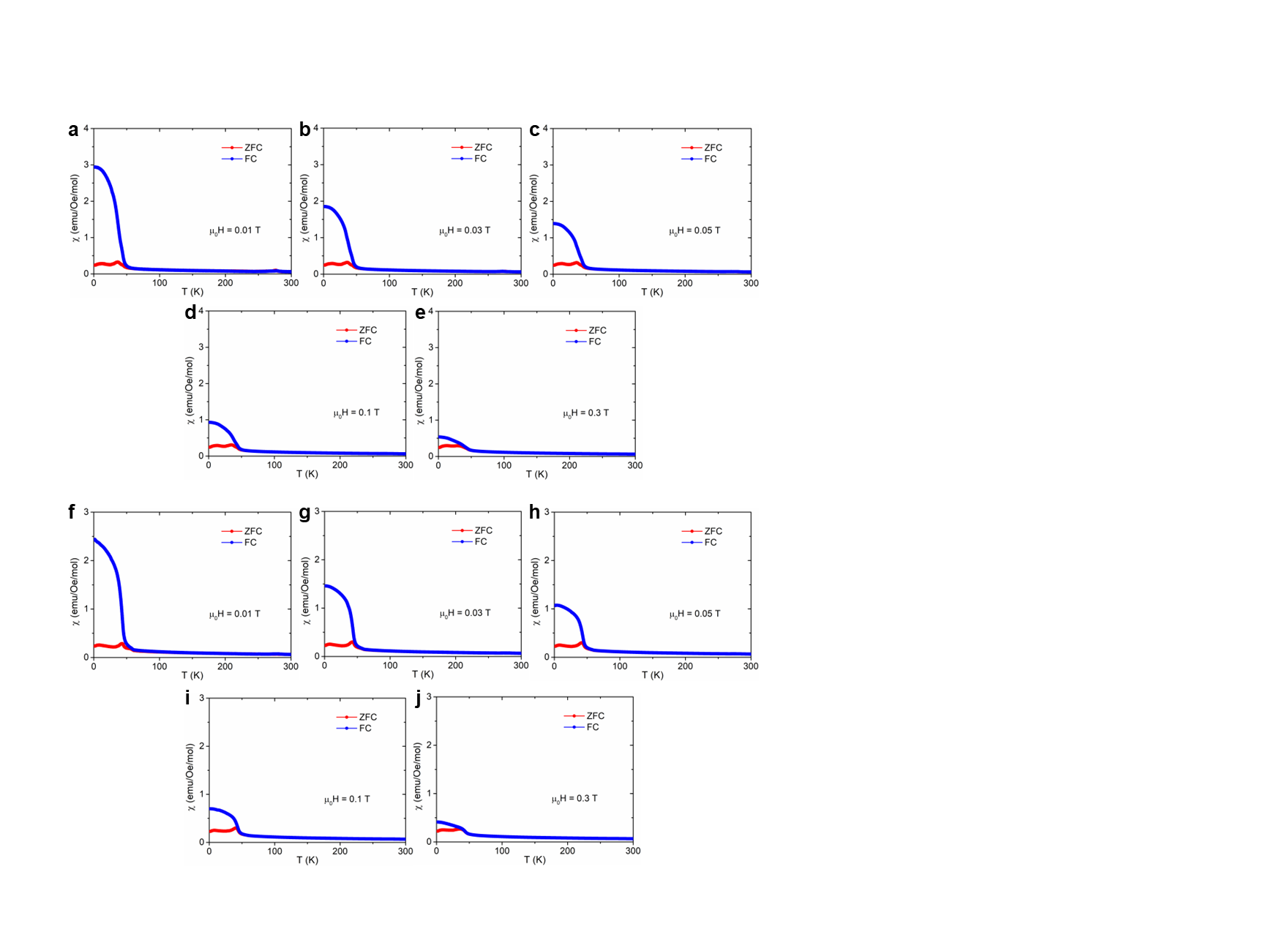
**Figure S2.** Temperature-dependent magnetic susceptibility with both ZFC and FC modes under different magnetic fields of **(a-e)** KSb_3_Mn_9_O_19_ and **(f-j)** RbSb_3_Mn_9_O_19_.


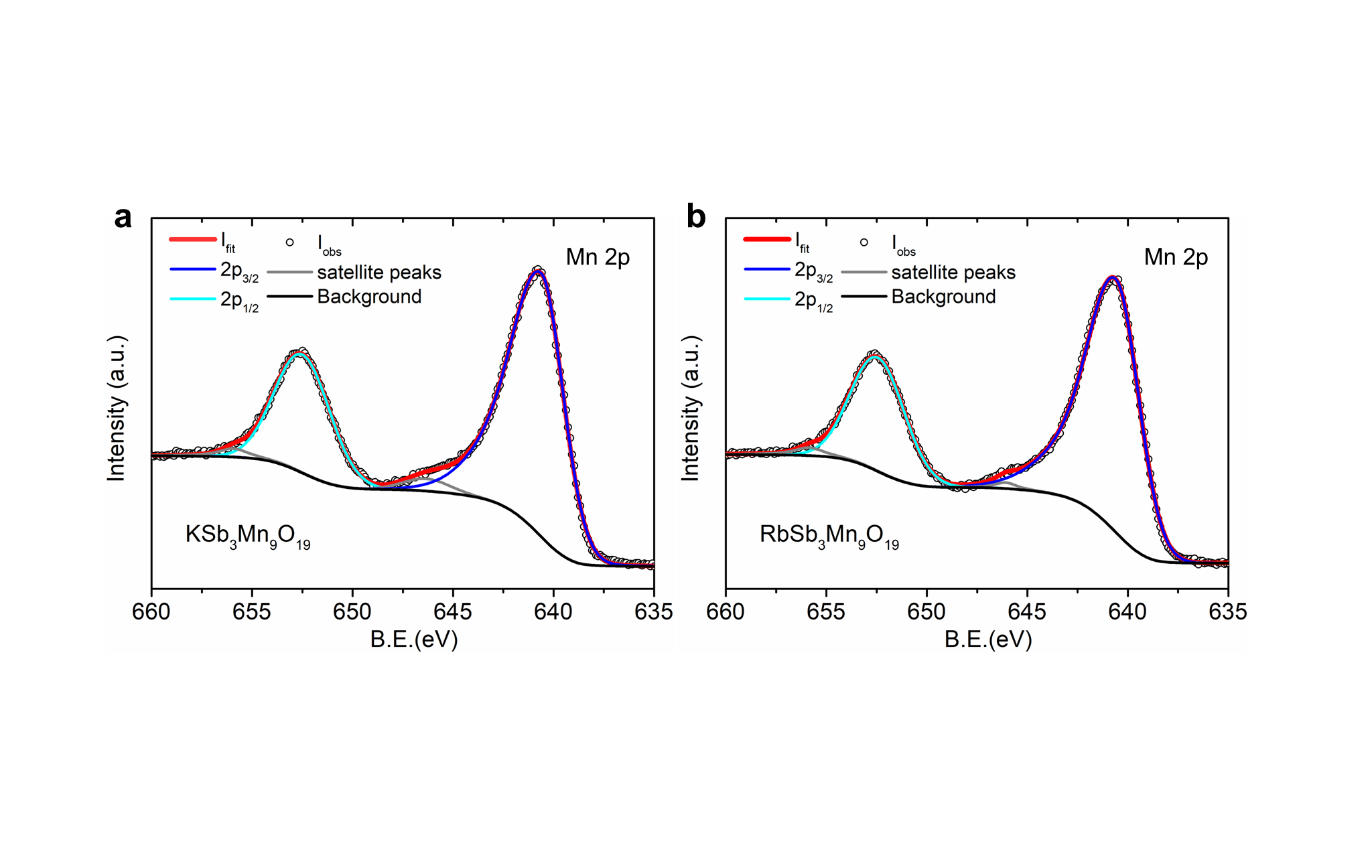
**Figure S3.** XPS spectra of Mn 2p orbitals for **a.** KSb_3_Mn_9_O_19_ and **b.** RbSb_3_Mn_9_O_19_.



**Figure S4.** Powder X-ray diffraction patterns of KSb_3_Mn_9_O_19_ with Mn_2_O_3_ as starting material.


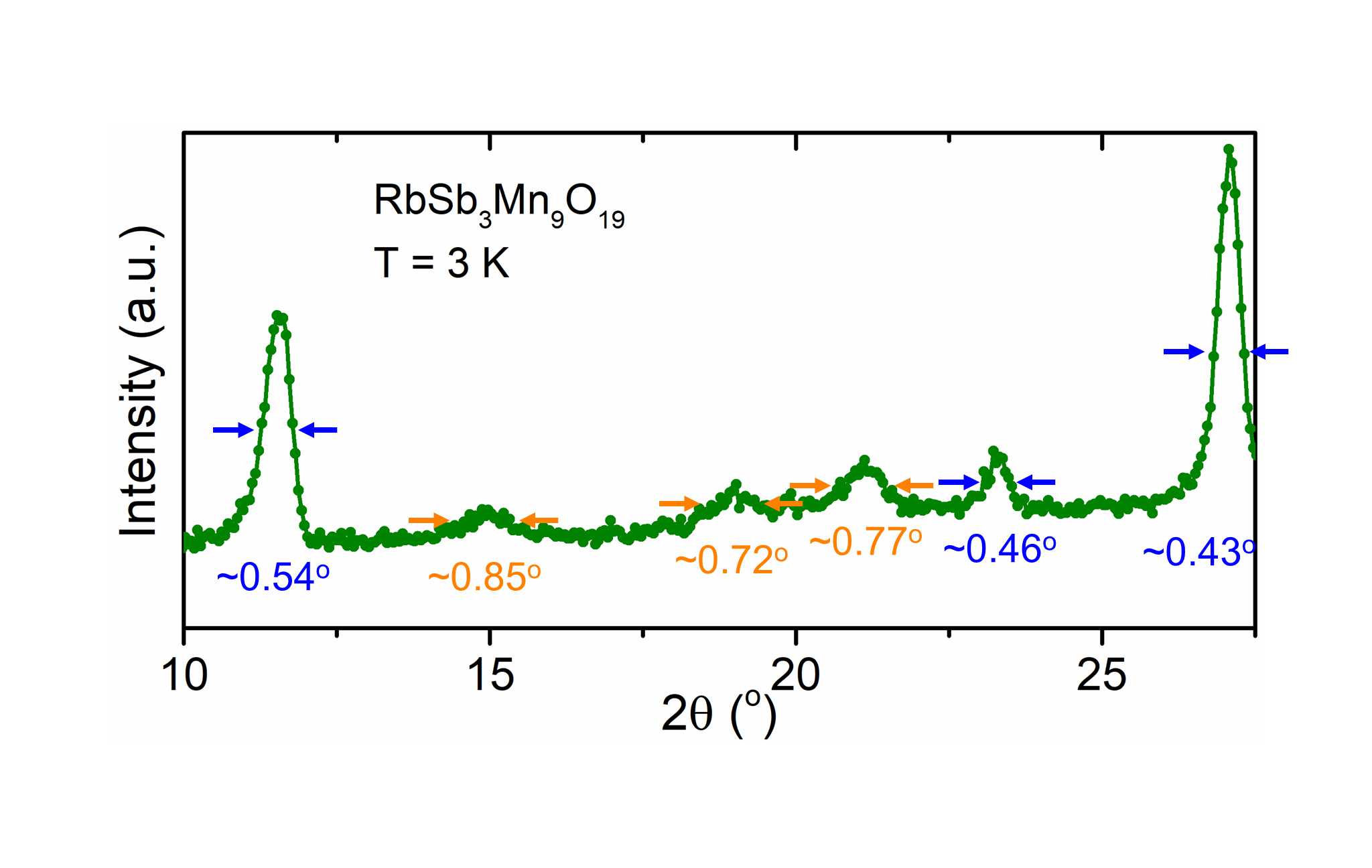
**Figure S5.** The peak width at low 2θ of RbSb_3_Mn_9_O_19_. The nuclear peaks and magnetic peaks and their full width at half maximum (FWHM) are marked by blue and orange, respectively.


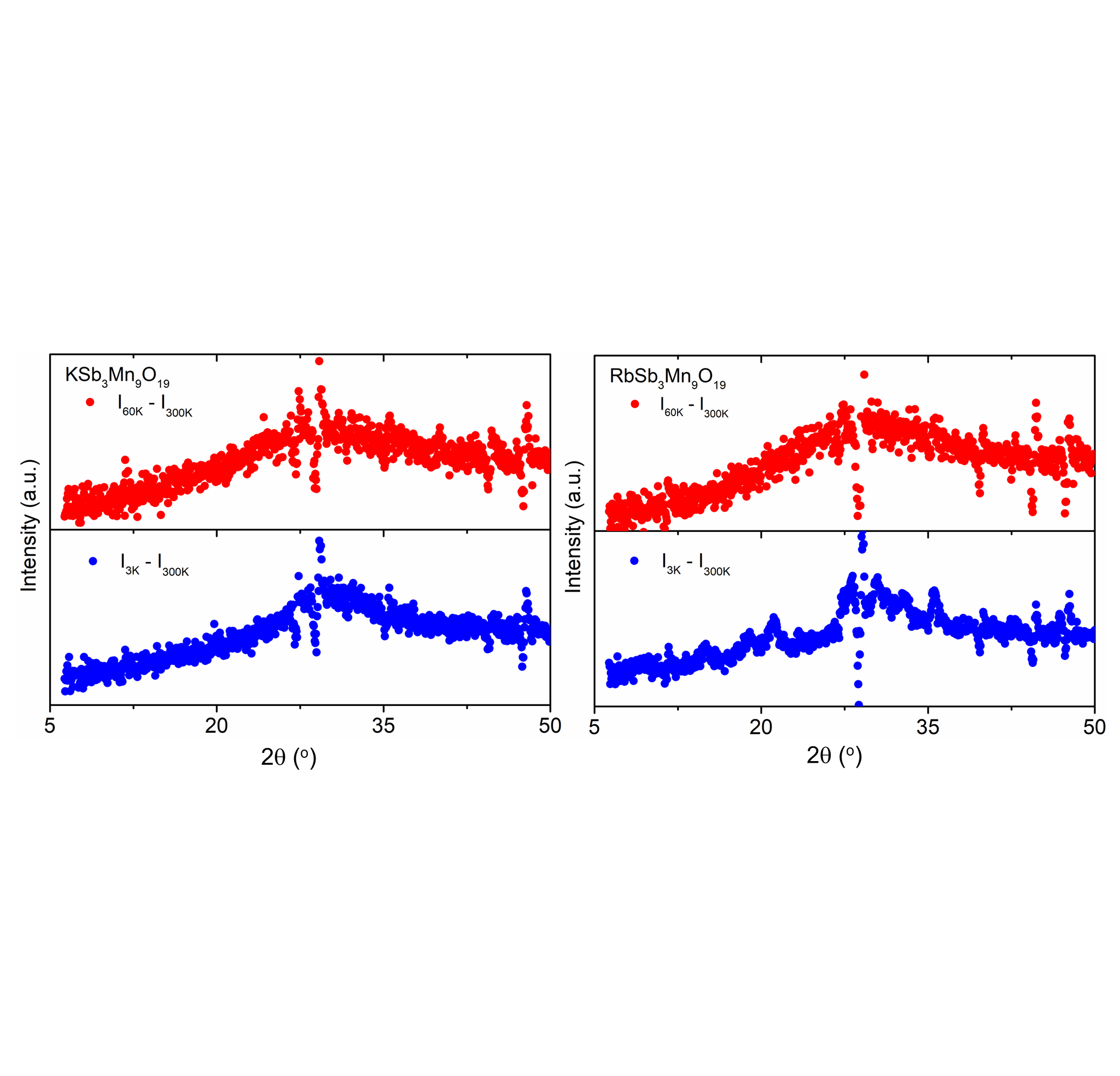
**Figure S6.** Evolution of diffuse scattering in ASb_3_Mn_9_O_19_ represented by subtracting the neutron powder diffraction pattern at 300 K from that of 60 K, as well as subtracting the pattern at 300 K from that of 3 K.
